# Sema3d^hi^ Fibroblasts Promote Acute Kidney Injury Fibrotic Progression Through Confining Endothelial Cell Migration

**DOI:** 10.7150/ijbs.124971

**Published:** 2026-01-01

**Authors:** Qiuyu Xie, Xiaohong Xin, Weijian Yao, Zehua Li, Yuanyuan Ma, Lei Qu, Yiyi Ma, Chengang Xiang, Suxia Wang, Gang Liu, Ying Chen, Li Yang

**Affiliations:** 1 Renal Division, Peking University Institute of Nephrology, Beijing Key Laboratory of Precision Medicine and New-drug/Equipment Development for Severe Kidney Disease, Key Laboratory of Renal Disease-Ministry of Health of China, Key Laboratory of CKD Prevention and Treatment (Peking University)-Ministry of Education of China, Research Units of Diagnosis and Treatment of Immune-mediated Kidney Diseases-Chinese Academy of Medical Sciences, Peking University First Hospital, Xishiku Street #8, Beijing, 100034, China.; 2 Laboratory of Animal Facility, Peking University First Hospital, Beijing, 100034, China.; 3 Laboratory of Electron Microscopy, Pathological Center, Peking University First Hospital, Beijing, 100034, China.

**Keywords:** acute kidney injury, fibroblast, endothelial cell, angiogenesis, migration, Sema3d

## Abstract

Acute kidney injury (AKI) often leads to incomplete recovery of renal function, progressing to chronic kidney disease (CKD). Key pathological features in the AKI-CKD transition include microvascular rarefaction and fibrosis. However, the direct effects of activated fibroblasts on microvasculature and endothelial cells remain unclear. We constructed a single-cell RNA sequencing (scRNA-seq) database from unilateral ischemia reperfusion injury (uIRI) mouse model and identified five heterogeneous fibroblast subpopulations, with C0-Sema3d^hi^ fibroblasts significantly increasing post-injury and correlating with reduced endothelial cells. Conditioned medium from Sema3d^hi^-NRK49F cells inhibited focal adhesion formation and induced cytoskeletal collapse in human umbilical vein endothelial cells (HUVECs), preventing migration and angiogenesis. Mechanistically, Sema3d^hi^ fibroblasts secrete Sema3d, activating the endothelial Plexin D1 receptor, leading to Arf6 activation and integrin β1 internalization, thus suppressing endothelial function. Systemic administration of Sema3d-shRNA using adeno-associated virus serotype 9 (AAV9) effectively reduced Sema3d levels and significantly alleviated renal fibrosis in mice. The presence of SEMA3D^+^ fibroblasts was confirmed by analyzing human scRNA-seq data and through immunofluorescence staining of kidney sections from patients with kidney diseases. This study reveals new target for mitigating renal fibrosis and microvascular loss, suggesting that targeting the Sema3d signaling pathway may provide a novel strategy for preventing AKI fibrotic progression.

## Introduction

Acute kidney injury (AKI) is a clinical syndrome characterized by a rapid decline in kidney function due to various etiologies, and it has emerged as a significant global public health issue 1. The most common type of AKI is ischemic AKI, caused by inadequate renal perfusion 2. Early stages of AKI often lack obvious symptoms, and patients frequently present with complex clinical conditions, leading to delayed diagnosis 3. Furthermore, there is a lack of effective treatments to mitigate renal tissue damage and promote kidney recovery, resulting in limited improvement in the prognosis of AKI patients 4. Although the initial injury mechanisms of AKI vary depending on the cause, studies have shown that almost all types of AKI carry a risk of progression to chronic kidney disease (CKD) 5.

After AKI, not only do renal tubular epithelial cells initiate repair programs, but levels of pro-angiogenic factors also rise within the kidney to promote angiogenesis 6-8. During this process, pericytes first detach from the vascular wall, followed by matrix metalloproteinase (MMP)-mediated degradation of the basement membrane, which loosens connections between endothelial cells. Integrins on the surface of endothelial cells mediate migration of endothelial cells to the extracellular matrix (ECM) surface, facilitating endothelial cells exposure to angiogenic factors 9. The expression of the Notch ligand Delta-like protein 4 (DLL4) is upregulated in endothelial cells, resulting in tip cell formation that guides new vessel growth. Tip cells activate the Notch signaling pathway in adjacent stalk cells, promoting their proliferation, extension of stalks, and lumen formation 10. Myeloid bridge cells facilitate the fusion of new vessels with other vascular branches, initiating blood flow perfusion 9.

During the formation of new blood vessels, constructing a proper vascular network is crucial for their normal physiological function. Semaphorins act as important environmental guidance molecules for endothelial cells, defining the boundaries of specific vascular regions 9, 11. Semaphorins are a highly conserved protein family, divided into seven classes. In vertebrates, semaphorins (classes 3 to 7) are typically secreted or transmembrane proteins that bind to corresponding plexin or neurophilin co-receptors 12, 13. During the separation of the primitive venous plexus in developing pulmonary veins, the expression of Sema3d restricts the migration of pulmonary vein endothelial cells. Subsequently, Sema3d acts as an endothelial cell repellent, guiding the correct connection of pulmonary veins to the left atrium 11. Additionally, many semaphorins have been reported to inhibit tumor angiogenesis during tumor formation 14-17.

Although angiogenic response is key to the repair of capillary rarefaction, the renal microvasculature is thought to have a limited capacity for repair 18, 19. One reason may stem from the inherent properties of renal endothelial cells, such as low proliferative capacity and susceptibility to endothelial to mesenchymal transition (EndoMT), another reason may due to the transformation of pericytes into myofibroblasts, leading to disruption of microvascular structure and stability 20-22. Furthermore, fibroblasts become activated after injury, undergo proliferation, migration, and increased ECM synthesis, causing excessive ECM deposition in the interstitium, consequently compressing the capillaries and aggravating capillary rarefaction 23. However, whether the extensive presence of fibroblasts in the interstitium directly impacts the regeneration of renal microvasculature during the AKI-CKD transition, inhibiting new blood vessel formation, requires further investigation.

In this study, we developed a mouse model of AKI-CKD induced by unilateral kidney ischemia-reperfusion (uIRI) and established a single-cell RNA sequencing (scRNA-seq) database of mouse whole kidney cells. By analyzing the scRNA-seq data of the fibroblast population, we identified a subgroup of fibroblasts that emerge after injury, characterized by high expression of the *Sema3d* gene, which is significantly different from classical myofibroblasts. Further mechanistic exploration revealed that Sema3d^hi^ fibroblasts secrete Sema3d to act on surrounding endothelial cells, inhibiting their migration and thereby impeding angiogenesis, exacerbating microvascular rarefaction, thus promoting renal fibrosis. Systemic administration of Sema3d-shRNA via adeno-associated virus serotype 9 (AAV9) effectively reduced Sema3d levels in the kidneys, leading to a significant alleviation of renal fibrosis following uIRI injury in mice. The presence of SEMA3D^+^ fibroblasts was also detected in human scRNA-seq dataset and through immunofluorescence staining of kidney sections from patients with kidney diseases. Our study confirms the direct impact of fibroblasts on endothelial cell migration and angiogenesis post-AKI, providing a potential new target for reducing microvascular loss and renal fibrosis after AKI.

## Materials and Methods

### Mice

Male C57BL/6J mice aged 10-12 weeks were purchased from Beijing Sibeifu Co., Ltd. All animals were housed in SPF level animal facilities under controlled environmental conditions with a 12-hour light/dark cycle. All animal procedures were approved by the Animal Welfare and Ethics Committee of Peking University First Hospital (Approval Number: J2022125), and were conducted in accordance with the guidelines for the care and use of laboratory animals.

### uIRI model

Mice were anesthetized with isoflurane and placed on a heating pad to maintain a body temperature of 37°C. A left flank incision was made to expose the left kidney. In the uIRI group, the left renal pedicle was occluded with a microvascular clamp for 45 min, after which the clamp was removed to allow reperfusion, indicated by the return of normal kidney color. In the sham group, the same incision was made without occlusion. The surgical site was closed in layers, and postoperative monitoring was conducted to assess recovery and detect potential complications.

### Preparation of whole kidney single cell suspension for scRNA sequencing

For the control mice and those that post uIRI surgery at various time points (1, 3, 5, 7, 10, 17 and 30 days, n=6 for each time points), the mice were anesthetized, and 10 mL of phosphate-buffered saline (PBS) was perfused through the left ventricle. The left kidney was then harvested for the preparation of single-cell suspension followed previously established protocols 24. Briefly, the kidneys were cut into 1 mm³ fragments on ice and incubated in 5 mL of digestion buffer containing 0.25 mg/mL Liberase™ TH (Thermolysin High) (Roche, Indianapolis, IN, USA) and 50 μg/mL DNase I (NEB, Ipswich, MA, USA) at 37 °C for 30 min. After digestion, the resulting cell suspension was filtered through a 40 μm cell strainer, centrifuged at 400 × g at 4 °C for 5 min, and resuspended in 500 μL of pre-chilled PBS containing 0.04% BSA. Dead cells were removed using the Dead Cell Removal Kit (Miltenyi Biotec, Germany, 130-090-101), and viable cells were collected.

### scRNA-Seq by 10×Genomics, data processing and quality control

ScRNA-seq by 10×Genomics was performed as previously described 25. Briefly, whole kidney cells were resuspended to a final cell concentration of 700~1200 cells/μL with more than 85% viability, as determined by Countess™ II (Thermo Fisher Scientific, Waltham, MA, USA). 8000 to 12000 cells were captured in droplets (10× Genomics Chromium Single Cell 3ʹ Reagent Kits;10× Genomics, Pleasanton, CA, USA). After the reverse transcription, fifty nanograms of amplified cDNA was fragmented, end-repaired, and sequenced on an Illumina platform (Illumina, San Diego, CA, USA) using 150 paired-end reads at a coverage of 40000 mean reads per cell. Raw sequencing reads were processed using Cell Ranger (v6.0.1, 10× Genomics) with the mouse reference genome (mm10). Quality control metrics were applied to filter low-quality cells and genes. Cells with fewer than 200 or more than 5,000 detected genes, and those with mitochondrial gene expression exceeding 50% were excluded from analysis. Genes expressed in fewer than 3 cells were also filtered out. Doublets were identified and removed using DoubletFinder (v2.0.3).

### Data normalization and dimensionality reduction

Filtered gene expression matrices were normalized using the SCTransform method implemented in Seurat (v4.1.0). Principal component analysis (PCA) was performed on the top 3,000 highly variable genes. The first 30 principal components were used for downstream analysis. Uniform Manifold Approximation and Projection (UMAP) was applied for two-dimensional visualization of the data.

### Cell clustering and annotation

Unsupervised clustering was performed using the shared nearest neighbor (SNN) graph-based clustering algorithm implemented in Seurat. A k-nearest neighbor (KNN) graph was first constructed using the top 30 principal components, followed by SNN graph construction with default parameters. Clusters were then identified using the SNN modularity optimization algorithm with a resolution parameter of 0.3. Cell type annotation was conducted based on the expression of canonical marker genes and comparison with published single-cell kidney atlases. Manual curation was performed to ensure accurate cell type assignment.

### Hierarchical sub-clustering analysis

To dissect the heterogeneity within stromal cell populations, we performed hierarchical sub-clustering analysis. First, mesenchymal cells identified in the initial clustering were extracted and subjected to sub-clustering analysis using SNN graph-based clustering with a resolution of 0.3, revealing distinct mesenchymal subpopulations. Subsequently, fibroblast populations identified within the mesenchymal sub-clusters were further extracted and re-clustered independently with a resolution of 0.4 to characterize fibroblast subtypes. Each level of sub-clustering involved re-normalization, scaling, principal component analysis, and UMAP visualization to ensure optimal resolution of cellular heterogeneity.

### Differential gene expression analysis

Differential gene expression analysis between cell types and experimental conditions was performed using the Wilcoxon rank-sum test implemented in Seurat. Genes with an adjusted *p*-value < 0.05 and log_2_ fold change > 0.25 were considered significantly differentially expressed. Multiple testing correction was applied using the Bonferroni method.

### Heatmap generation and visualization

Differential gene expression heatmaps were generated using the ComplexHeatmap package in R. Genes with significant differential expression (adjusted *p*-value < 0.05 and log_2_ fold change > 0.5) across cell types were selected for visualization. Expression values were z-score normalized and hierarchically clustered using Euclidean distance and complete linkage. Color scales were applied to represent expression levels from low (blue) to high (red).

### Violin plot analysis

Violin plots were generated using the VlnPlot function in Seurat to visualize gene expression distributions across cell populations. Each violin plot displays the kernel density estimation of gene expression levels, with the width of the violin representing the density of cells at each expression level. Median expression values are indicated by horizontal lines within each violin.

### Cluster proportion analysis

Cluster composition analysis was performed to assess the relative abundance of each cell cluster across different experimental conditions. Cell counts for each cluster were calculated and converted to percentages of the total cell population. Stacked bar charts were generated using ggplot2 to visualize the proportional changes in cluster composition.

### Gene ontology enrichment analysis and visualization

Functional enrichment analysis was performed using the clusterProfiler package (v4.0.5) in R. For each cluster, differentially expressed genes (adjusted *p*-value < 0.05 and log_2_ fold change > 0.25) were identified and subjected to GO enrichment analysis for biological processes. The enrichGO function was used with the org.Mm.eg.db annotation database for mouse. Enriched GO terms with adjusted *p*-value < 0.05 (Benjamini-Hochberg correction) were considered statistically significant. Results were visualized using violin plots generated with the ggplot2 package (v3.3.5), where the width of each violin represents the density distribution of enrichment scores across different clusters, allowing for comparison of GO term enrichment patterns between cell populations.

### Trajectory analysis and pseudotime ordering

Pseudotime analysis was conducted using Monocle3 (v1.0.0) to reconstruct developmental trajectories and infer cell state transitions. Single-cell expression data were converted to a cell_data_set object, and the top 1,000 most variable genes were selected for trajectory construction. Dimensionality reduction was performed using UMAP, followed by graph-based clustering and trajectory learning using the learn_graph function. Pseudotime values were calculated using the order_cells function, with the root of the trajectory manually selected based on biological knowledge. Cells were ordered along the inferred trajectory, and the results were visualized with cells colored by cluster identity to reveal differentiation pathways.

### Ligand-receptor interaction analysis

Cell-cell communication analysis was performed using CellChat v1.1.3. Ligand-receptor pairs were identified from the CellChatDB database, which contains manually curated interactions from literature and public databases. For each cell type pair, the number of significant ligand-receptor interactions was calculated based on expression criteria (≥10% of cells expressing the gene with average log-normalized expression >0.1) and statistical significance (*p*<0.05, permutation test with 100 iterations).

Communication probabilities between cell types were computed using the CellChat algorithm, which integrates expression levels, cell population sizes, and interaction confidence scores. The interaction strength was calculated as the product of average ligand expression in sender cells and average receptor expression in receiver cells, weighted by the proportion of expressing cells in each population. Heatmap visualization was generated using the ComplexHeatmap package v2.8.0, with hierarchical clustering applied using Euclidean distance and complete linkage method.

For detailed ligand-receptor network visualization, dot plots were created using the DotPlot function in Seurat v4.0.3, where dot size represents the percentage of cells expressing each gene within each cell type, and color intensity indicates the scaled average expression level. Only ligand-receptor pairs with significant communication probabilities (*p*<0.05) were included in the final analysis. Statistical significance was assessed using permutation tests implemented in CellChat, comparing observed interaction strengths to null distributions generated by randomly permuting cell type labels.

### Immunofluorescence staining

Mouse kidney samples were fixed in 10% formalin at room temperature for 24 hours, followed by dehydration, clearing, paraffin infiltration, and embedding. Paraffin sections (2µm) were deparaffinized in xylene and then hydrated through a gradient of ethanol (100%, 95%, 80%) and washed three times with PBS for 3 min each time. Antigen retrieval was performed by high-pressure heating in EDTA buffer (pH 9.0), followed by natural cooling to room temperature. The sections were blocked at room temperature with blocking solution for 30 min, then incubated overnight at 4°C with primary antibodies, including Sema3d (MG749917S, Abmart, China), Cd31 (#77699T, Cell Signaling Technology, USA), Pdgfrα (AF1062, R&D, USA), Plexin D1 (DF13502, Affinity, China), α-SMA (Abcam, ab124964, USA) and GFP (#2956, Cell Signaling Technology, USA), followed by incubation with fluorescent secondary antibodies (Jackson ImmunoResearch, USA) at room temperature for 1 hour. Finally, the sections were mounted using DAPI-containing mounting medium.

### Cell culture

NRK-49F cells (National Collection of Authenticated Cell Cultures, Shanghai, China) were maintained in DMEM (TransGen Biotech, China) supplemented with 5% FBS (Gibco, USA), and 1 × penicillin-streptomycin (Gibco, USA). HUVECs (ScienCell, USA) were cultured in endothelial cell medium (ECM, ScienCell, USA). All cultures were incubated at 37 °C in a humidified atmosphere containing 5% CO₂. The culture medium was refreshed approximately every 48 hours. Cells were passaged when they reached 80-90% confluency.

### Lentiviral transduction

When NRK-49F cells reached 50-70% confluency, lentiviral transduction was performed. Lentiviruses containing either the Sema3d-overexpressing plasmid or a control vector plasmid (Shanghai Genechem Co., Ltd.) were added to complete medium at a multiplicity of infection (MOI) of 50. Then, 1 × transfection reagent (Shanghai Genechem Co., Ltd.) was added to the viral solution to prepare the transfection working solution. After 16-24 hours, the transfection solution was removed and replaced with fresh complete medium for continued culture. Once the cells reached 90-100% confluency, the medium was switched to complete medium containing 8 μg/mL puromycin. Subsequently, the cells were maintained in complete medium supplemented with 4 μg/mL puromycin.

### Enzyme-linked immunosorbent assay (ELISA)

The levels of Sema3d in conditioned medium of NRK-49F cells were measured using Sema3d Quantification ELISA Kit (AF21484-A, Aifang, China). The stock standard solution was subjected to serial dilutions to generate a concentration gradient. All concentrations of the standard and the samples were added into the antibody-coated plate and incubated at 37°C for 30 min. Wash the plate with washing buffer 5 times for 30 sec each time. Then enzyme-labeled antibody was added to each well and incubated at 37°C for 30 min. Wash the plate 5 times. Chromogenic substrate was added to each well and incubated in the dark at 37°C for 10 min. Stop solution was added to terminate the reaction. Absorbance at 450 nm of each well was measured. Curve Expert 1.4 was used to fit the standard curve and calculate the samples concentrations.

### Fibroblast and HUVEC repulsion assay

Cells (1.5 × 10^4^) from both NRK49F-OE and NRK49F-Vector groups were seeded onto HUVEC monolayers with 80% confluency. After 72 hours, fluorescence and bright field images were captured by microscope (Echo Laboratories, USA). The distances between fibroblasts and surrounding HUVECs were analyzed with ImageJ software and calculated by the following formula:

Distance of fibroblast to surrounding HUVECs = The sum of distances between fibroblast to its surrounding HUVECs / Total number of HUVECs around the fibroblast

### Endothelial cell wound healing assay

When HUVECs reached 100% confluency, a linear scratch wound was created in the cell monolayer using a sterile pipette tip. The detached cells were subsequently washed away with PBS. Conditioned medium from the normal NRK-49F, NRK49F-Vector, or NRK49F-OE groups was then added to the respective HUVEC cultures. After a 12-hour incubation at 37 °C in a 5% CO₂ atmosphere, images of the HUVECs were captured. The migration rate was analyzed using ImageJ software and calculated as follows:

Cell migration rate = (Initial scratch area - Scratch area after 12 h) / Initial scratch area

### Endothelial cell chemotactic migration assay

Equal number of NRK-49F, NRK49F-Vector and NRK49F-OE were seeded in the lower chamber of the Transwell system (pore size 8 µm, Corning, USA). Once the fibroblasts reached 80% confluency, the medium was replaced with ECM containing 20% FBS. HUVECs (1 × 10^5^) were seeded in the upper chamber of each Transwell group and allowed to co-culture for 48-72 hours. After this, HUVECs in the upper chamber were fixed with 4% paraformaldehyde for 15 min, followed by staining with 0.1% crystal violet solution for 20 min. HUVECs migrated through the Transwell chambers were observed under microscope and the number was counted using ImageJ software.

### Angiogenesis assay

The bottoms of pre-cooled culture plates were uniformly coated with Matrigel (AC-M082704, Acro Biosystems, China) and incubated at 37 °C for 1 hour to allow solidification. HUVECs (2 × 10^4^) were resuspended in conditioned media from the NRK-49F, NRK49F-Vector, or NRK49F-OE groups and were seeded into the Matrigel-coated wells, then cultured for 6 hours. After incubation, images of the vascular structures were captured, and ImageJ software was utilized to analyze the number of vascular structures in each field.

### Immunocytochemistry

HUVECs grown on coverslips were stimulated with fibroblast-conditioned medium or recombinant Sema3d protein (9386-S3-025, R&D, USA). Cells were fixed with 4% paraformaldehyde for 20 min at room temperature. Subsequently, the cells were treated with 0.3% Triton X-100 for 10 min and blocked in blocking solution for 30 min at room temperature. The coverslips were then incubated overnight at 4 °C with primary antibodies, including p-FAK (#3283, Cell Signaling Technology, USA) and vinculin (ab129002, Abcam, USA), followed by incubation with fluorescent secondary antibodies (Jackson ImmunoResearch, USA) at room temperature for 1 hour. FITC-conjugated phalloidin was added for 30 min at room temperature to stain the cytoskeleton. Coverslips were mounted using a DAPI-containing mounting medium.

### Integrin internalization assay

HUVECs were seeded on coverslips coated with fibronectin and cultured until reaching approximately 50% confluency. The serum was then removed, and the cells were starved overnight. Anti-β1 integrin antibody (sc-18887, Santa Cruz, USA) was diluted to a final concentration of 5 µg/mL in DMEM containing 0.01% BSA and pre-cooled at 4°C. This solution was added to the HUVECs and incubated on ice for 30 min to label cell surface integrins. The cells were washed twice on ice with cold 0.01% BSA-DMEM to remove excess antibody. HUVECs were then stimulated with fibroblast-conditioned medium or recombinant Sema3d protein. One group was immediately fixed with 4% paraformaldehyde, while another group was treated with an acid washing buffer (pH 2.2, 100 mM glycine, 20 mM magnesium acetate, 50 mM KCl) for 60 sec, followed by two washes with PBS, each for 2 min, and then fixed with 4% paraformaldehyde. The cells that underwent acid washing buffer were treated with 0.3% Triton at room temperature for 10 min, while the immediately fixed cells were not treated. After incubation with fluorescent secondary antibody for 1 hour at room temperature, the coverslips were mounted using a DAPI-containing mounting medium.

### Arf6 activation assay

HUVECs with 100% confluency were stimulated for 6 min with fibroblast-conditioned medium. Following stimulation, the plates were placed on ice, and the medium was aspirated. The cells were washed twice with PBS. Cell lysis was performed using lysis buffer containing protease inhibitors, according to the instructions of the Arf6 Pulldown Activation Assay Kit (BK033-S, Cytoskeleton, USA). The lysate was centrifuged at 10,000 × g for 1 min at 4 °C, and the supernatant was collected. For quantification, 10 µL of the cell lysate was analyzed using a BCA protein assay. A total of 20 µg of protein was reserved for western blot analysis to detect total Arf6 levels. For the pull-down assay, 800 µg of total protein was mixed with 20 µL of GGA3-PBD agarose beads and incubated with gentle rotation at 4 °C for 1 hour. The GGA3-PBD agarose beads were then collected by centrifugation at 5,000 × g for 2 min at 4 °C and washed twice with the provided washing buffer. Each sample was resuspended in 20 µL of 2× Laemmli loading buffer and heated at 100 °C for 5 min. The samples were subsequently centrifuged at 10,000 × g for 2 min at 4 °C, and the supernatants were collected for detection by western blot analysis.

### Quantitative polymerase chain reaction (qPCR) analysis

RNA was extracted according to the instructions of the RNA extraction kit (ER501-01-V2, TransGen Biotech, China), and the extracted RNA was reverse transcribed into cDNA using a reverse transcription kit (AU-341-02-V2, TransGen Biotech, China). Then qPCR experiments were conducted using qPCR Kit (RR820, Takara, Japan). Quantification was performed using the 2^-ΔΔCt^ method, and normalized according to the expression of the reference gene *Gapdh*. The primer sequences are listed in Table S1.

### Western blotting

RIPA containing protease inhibitors was used to lyse tissues or cells, and the supernatant was collected after centrifugation to obtain the protein. The protein was mixed with loading buffer and heated at 100°C for 10 min, then loaded onto SDS-PAGE gel. Electrophoresis was performed at 120V for 50 min. The gel and a PVDF membrane were electrophoretically transferred at 350mA at 4°C for 35 min. The membrane was then blocked at room temperature for 1 hour with TBST containing 5% non-fat milk. The membrane was covered with primary antibodies, including Flag (F1804, Merck, USA), GFP (ab183734, Abcam, USA), Sema3d (MG749917S, Abmart, China), GAPDH (60004-1-Ig, Proteintech, China), β-actin (66009-1-Ig, Proteintech, China), p-FAK (#3283, Cell Signaling Technology, USA), FAK (#3285, Cell Signaling Technology, USA), Plexin D1 (DF13502, Affinity, China), Igf1 (ab9572, Abcam, USA), Pcna (ab152112, Abcam, USA), p-p65 (#3033, Cell Signaling Technology, USA), p65 (#8242, Cell Signaling Technology, USA), Tspan4 (NBP1-59438, Novus, USA), Itgb1 (ab30394, Abcam, USA), Col1α1 (ab270993, Abcam, USA), and incubated overnight at 4°C. The membrane was washed three times with TBST for 5 min each, then incubated with HRP-conjugated secondary antibodies (Beyotime, China) at room temperature for 1 hour. The membrane was washed three times with TBST for 5 min each, then reacted with Chemiluminescent substrate, and exposed in a GE Image Quant LAS4000 chemiluminescence imaging analysis system. Density was measured using ImageJ software.

### siRNA transfection

HUVECs were transfected when they reached 50-70% confluency. siRNA duplexes (si-NC-Cy3 and si-Plexin D1-Cy3) were diluted in serum-free medium and mixed thoroughly. RNAFit was added to the mixture, which was then vortexed for 10 seconds and incubated at room temperature for 10 minutes. The cell culture medium was replaced with fresh complete medium, and the prepared transfection complexes were evenly distributed across the dishes. Cells were subsequently cultured in a 37 °C, 5% CO₂ incubator. After 6 hours, the transfection medium was replaced with fresh complete medium.

### AAV9-Sema3d shRNA preparation and administration

Five plasmids were engineered, including ssAAV.CAG.EGFP.miR30shRNA 1-4 (mSema3d).WPRE3.SV40 (knockdown) and pcDNA3.1(+)-PGK.mCherry-P2A-mSema3d(NM_028882.4).bGHpA (overexpression). Each Sema3d-shRNA plasmid was co-transfected with the overexpression plasmid into HEK293 cells using PEI reagent. The efficiency of knockdown was assessed through rt-qPCR and immunofluorescence detection. shRNA2 exhibited the most significant knockdown efficiency.

The target plasmid was co-transfected with pHelper plasmids into HEK293T cells using PEI. Cells were cultured in DMEM with 10% FBS at 37 °C. Viral particles were harvested after 72 h from both cell lysates and media, purified through iodixanol gradient ultracentrifugation, and concentrated by ultrafiltration. Final preparations were quantified by rt-qPCR and standardized to 1 × 10¹³ GC/mL. Vectors were manufactured by PackGene Biotech (Guangzhou, China).

AAV9-EGFP-Sema3d shRNA and AAV9-EGFP were injected into the tail vein of mice at a dose of 5×10^11^ GCs per mouse on day 3 post uIRI surgery. Each group included six mice undergoing uIRI procedures and three mice undergoing sham procedures.

### Histology

Paraffin-embedded tissue sections were sequentially deparaffinized in xylene and rehydrated through a graded ethanol series. For Masson staining, the sections were stained with Weigert's iron hematoxylin for 5 minutes, followed by rinsing under running tap water and differentiating with 1% acid alcohol until the sections turned blue. Subsequently, the sections were stained with Ponceau-acid fuchsin solution for 10 minutes, counterstained with aniline blue for 5 minutes, and briefly immersed in 1% acetic acid for 30 seconds. For Sirius red staining, sections were stained in a picro-Sirius red solution for 60 minutes, immersed in absolute ethanol for 30 seconds. For fibronectin antibody staining, the sections were incubated with the primary antibody against Fibronectin (ab2413, Abcam, USA). Afterward, an HRP-conjugated secondary antibody (PV9001, Zhongshanjinqiao, China) was applied at 37 °C for 40 minutes. Then, a DAB chromogenic solution was applied to develop for 1-2 minutes. The sections were then stained with hematoxylin for 5 minutes, differentiated in 1% acid alcohol, and rinsed under tap water until the nucleus turned blue. By the end of above staining, the tissue sections were dehydrated through a graded ethanol series, cleared in xylene, and mounted with neutral balsam.

### Human scRNA-seq data analysis

Human scRNA-seq data were obtained from the Kidney Precision Medicine Project (KPMP) website, which has granted approval for our use of KPMP data in accordance with their guidelines. For our analysis, we selected the following KPMP samples: 34-10187, 29-10013, KRP461, 30-10034, 29-10008, and 3490. This dataset includes four disease samples (AKI and hypertensive CKD [H-CKD]) and two healthy control samples. The raw data were downloaded in h5ad format on October 11, 2025.

The raw data were processed using the standard KPMP pipeline and analyzed using the following software and packages: Python 3.9+, Scanpy 1.9.x, Harmonypy (for batch correction), NumPy, Pandas, SciPy, Matplotlib, Seaborn. Cell type annotation was performed based on the expression of canonical kidney cell type marker genes (Table S2). Expression levels of genes of interest (e.g., PLXND1, SEMA3D) were extracted from the normalized expression matrix. Cells were classified as "high expression" if the log-normalized expression exceeded a threshold of 1.0. Expression patterns were visualized using UMAP plots and split UMAP plots stratified by disease status.

### Multiplex immunohistochemistry of patient kidney samples

We selected three human kidney tissue samples for multiplex immunohistochemistry, including two samples from patients diagnosed with acute kidney disease (AKD) and chronic kidney disease (CKD), respectively, as well as one donor kidney used as a control (Table S3). All samples were obtained from Peking University First Hospital in 2025. The protocol concerning the use of patient samples was approved by the Biomedical Research Ethics Committee of Peking University First Hospital (approval number: 2017[1280]).

Patient kidney samples were deparaffinized and rehydrated through a series of xylene and alcohol washes. Antigen retrieval was performed in EDTA solution (pH 9.0) at 95-99 °C for 30 minutes. The tissue sections were incubated with a cocktail of primary antibodies, including PDGFRα (#5241, Cell Signaling Technology, USA), SEMA3D (HPA037522, Sigma, USA), PLEXIND1 (DF13502, Affinity, China), CD31 (ab281583, Abcam, USA). Following this incubation, sections were washed and incubated with secondary antibodies that were specific to the primary antibodies. Afterward, the sections were reacted with the TSA working solution (RC0086, Huilan Biotech, China) at room temperature for 5 minutes. Then the sections were subjected to heat-induced antibody elution in EDTA solution (pH 9.0). This cycle was repeated until all target antibodies had been labeled. Finally, the sections were mounted using a DAPI-containing mounting medium.

### Statistical analysis

Statistical analyses of scRNA-seq data were performed using R (v4.1.0). Data visualization was conducted using ggplot2 and other R packages. For other data, statistical analyses and graphing were performed using GraphPad Prism 8 (GraphPad, Chicago). Differences between groups were analyzed using t-test or one-way ANOVA. Correlation analysis was conducted using Pearson correlation analysis. *P* < 0.05 was considered statistically significant. Multiple testing corrections were applied where appropriate.

## Results

### Single-cell transcriptomic atlas of renal fibroblasts in mice following uIRI

**Figure 1:**
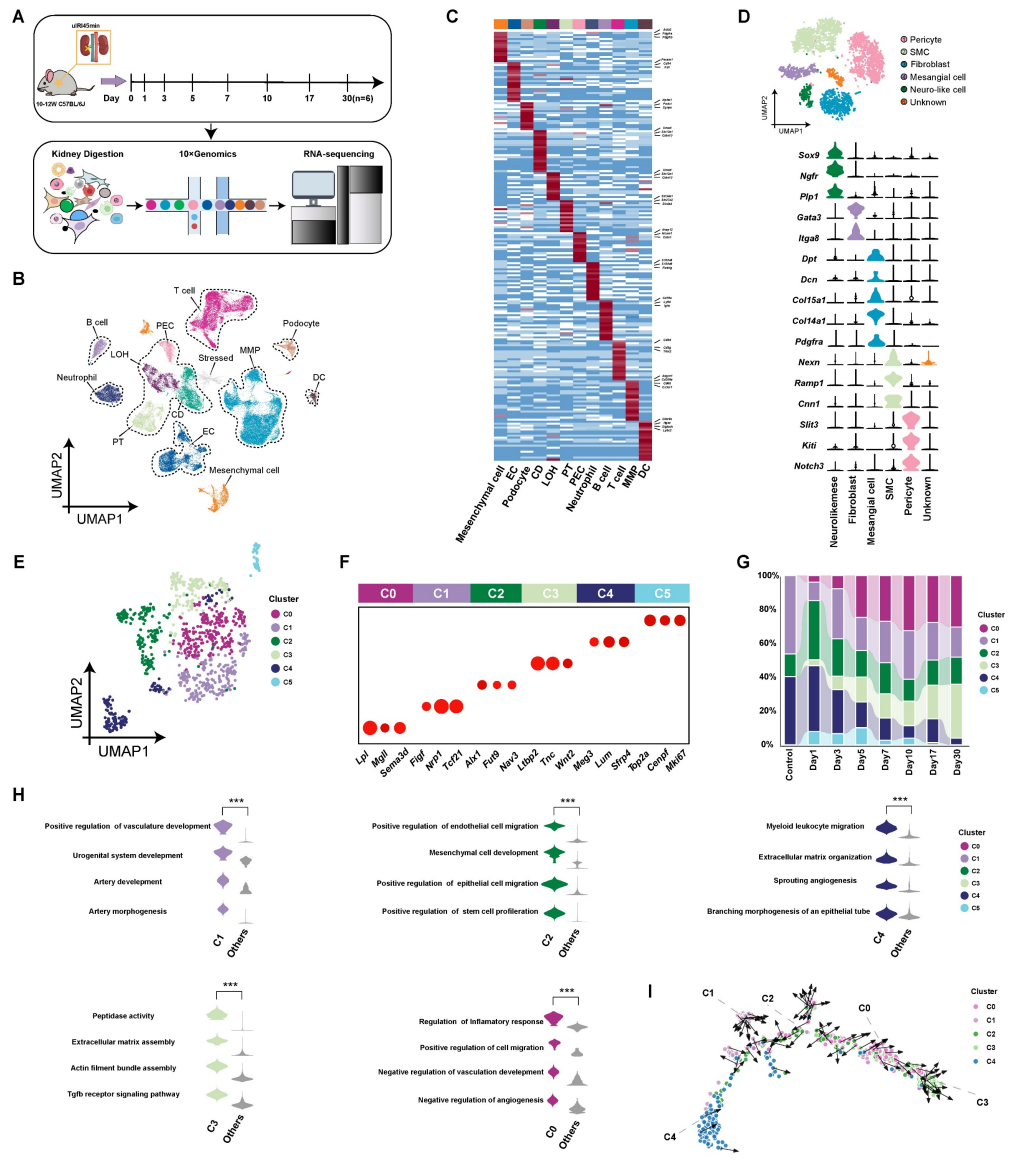
** Single-cell sequencing atlas of renal fibroblasts in uIRI mice. (A)** Overview of single-cell sequencing library construction from uIRI mice. **(B)** UMAP plot of scRNA-seq data from 78,250 kidney cells in control and uIRI mice. PT, proximal tubule; CD, collecting duct; LOH, loop of Henle; PEC, parietal epithelial cells; MMP, monocyte-macrophage; DC, dendritic cell; EC, endothelial cell. **(C)** Heatmap displaying expression levels of specific marker genes for cell compartments, with compartment types indicated. **(D)** UMAP plot of 6,398 renal mesenchymal cells from control and uIRI mice, color-coded by cell cluster, with types labeled. Violin plot shows expression levels of marker genes for each mesenchymal cluster. SMC, smooth muscle cell; Neuro-like mese, neural crest-like mesenchymal cells. **(E)** UMAP plot of 955 renal fibroblast cells from control and uIRI mice, color-coded by subcluster, with subcluster names labeled.** (F)** Bubble plot of marker gene expression levels for each fibroblast subcluster, with subcluster representation indicated. **(G)** Stacked bar plot showing the proportion of each fibroblast subcluster over time, color-coded by subcluster. **(H)** Violin plot of GOBP enrichment based on DEGs of fibroblast subclusters. **(I)** Pseudotime developmental trajectory of fibroblast subclusters post-uIRI.

We performed uIRI surgery for 45 min on male C57BL/6J mice aged 10-12 weeks, and subsequently collected kidney tissue samples at post-operative days 1, 3, 5, 7, 10, 17, and 30, as well as from control mice. We observed the pathological changes in renal tissues after AKI, including early cell apoptosis/necrosis and immune cell infiltration, then cell proliferation during the repair phase, and late-stage renal interstitial expansion and fibrosis after IRI (Figure S1). A scRNA-seq database of kidney cells throughout AKI-CKD transition was constructed (Figure 1A). After integrating the scRNA-seq data from the control group and the uIRI group, we obtained a total of 78,250 kidney cells that passed quality control. These kidney cells were classified into 26 clusters through unsupervised clustering. Based on the differentially expressed genes (DEGs) of these 26 cell clusters (Table S4), the kidney cells were annotated and categorized into 12 cell compartments according to their characteristics and functions, which mainly included epithelial cell partition (proximal tubule, loop of Henle, collecting duct, podocytes, and parietal epithelial cells), immune cell partition (monocyte-macrophage, T cells, B cells, dendritic cells, and neutrophils), endothelial cell partition, and mesenchymal cell partition (Figure 1B-C).

After comparing with literature 26-28 and public databases, 6,398 cells expressing high levels of *Pdgfrb*, *Pdgfra*, *Acta2*, and *Col1a1* genes were defined as mesenchymal cells. Subsequently, we conducted subclustering analysis on mesenchymal cells and obtained 6 subpopulations (pericytes, smooth muscle cells, fibroblasts, mesangial cells, neuro-like cells and unknown cells), of which 955 cells specifically highly expressing *Dpt*, *Dcn*, *Col15a1*, *Col14a1*, and *Pdgfra* genes were defined as typical fibroblasts 26, 29-33 (Figure 1D and Table S5). These fibroblasts were divided into 6 subpopulations (C0-C5) by unsupervised clustering analysis (Figure 1E). Among the subpopulations, C1-Tcf21^hi^ (Transcription factor 21, transcription factor), C2-Fut9^hi^ (Fucosyltransferase 9, secreted protein), and C4-Sfrp4^hi^ (Secreted frizzled-related protein 4, secreted protein) are resident kidney fibroblasts, while the C0-Sema3d^hi^ (Semaphorin 3D, secreted protein) and C3-Ltbp2^hi^ (Latent transforming growth factor beta-binding protein 2, extracellular matrix protein) appeared after injury (Figure 1F and G and Table S6). C5 represented the rapidly proliferating cell population, and so it was not included in subsequent analysis. The proportion of resident fibroblast subpopulations (C1, C2 and C4) decreased progressively over time after uIRI (Figure 1G). Conversely, C0-Sema3d^hi^ and C3-Ltbp2^hi^, which appeared after injury, gradually increased in proportion, and became the two main fibroblast subpopulations in kidneys in late stage of injury (Figure 1G).

To explore characteristics and functions of the subpopulations, DEGs of each subpopulation were subjected to gene ontology biological process (GOBP) enrichment analysis (Figure 1H). The resident fibroblast subpopulations were enriched for biological functions related to promoting tubular epithelial cell repair and angiogenesis. Additionally, the C4-Sfrp4^hi^ subpopulation was enriched for biological functions related to extracellular matrix assembly and leukocyte chemotaxis compared to other resident subpopulations. Among the subpopulations that appeared after injury, the C3-Ltbp2^hi^ subpopulation may present stronger contractile and pro-fibrotic characteristics, resembling traditionally defined myofibroblasts. The C0-Sema3d^hi^ is the largest subpopulation (24.7%) of fibroblasts throughout AKI-CKD process, being enriched for biological functions related to migration, inflammation regulation, and inhibition of angiogenesis. The developmental trajectory analysis of fibroblast subpopulations showed that during the AKI-CKD transition, fibroblasts transformed from resident subpopulations of C1-Tcf21^hi^, C2-Fut9^hi^, and C4-Sfrp4^hi^ to C0-Sema3d^hi^, and finally to the terminally differentiated C3-Ltbp2^hi^ myofibroblast subpopulation. (Figure 1I). Immunofluorescence staining of the C0 marker Sema3d with both the fibroblast marker Pdgfrα and the myofibroblast marker α-SMA on kidney tissues from uIRI mice at day 7 post-injury demonstrated partial co-localization (Figure S2). This finding suggests that the C0-Sema3d^hi^ fibroblasts may be in a transitional stage from fibroblasts to myofibroblasts.

### Enhanced interaction between fibroblasts and endothelial cells in the kidney following uIRI

**Figure 2:**
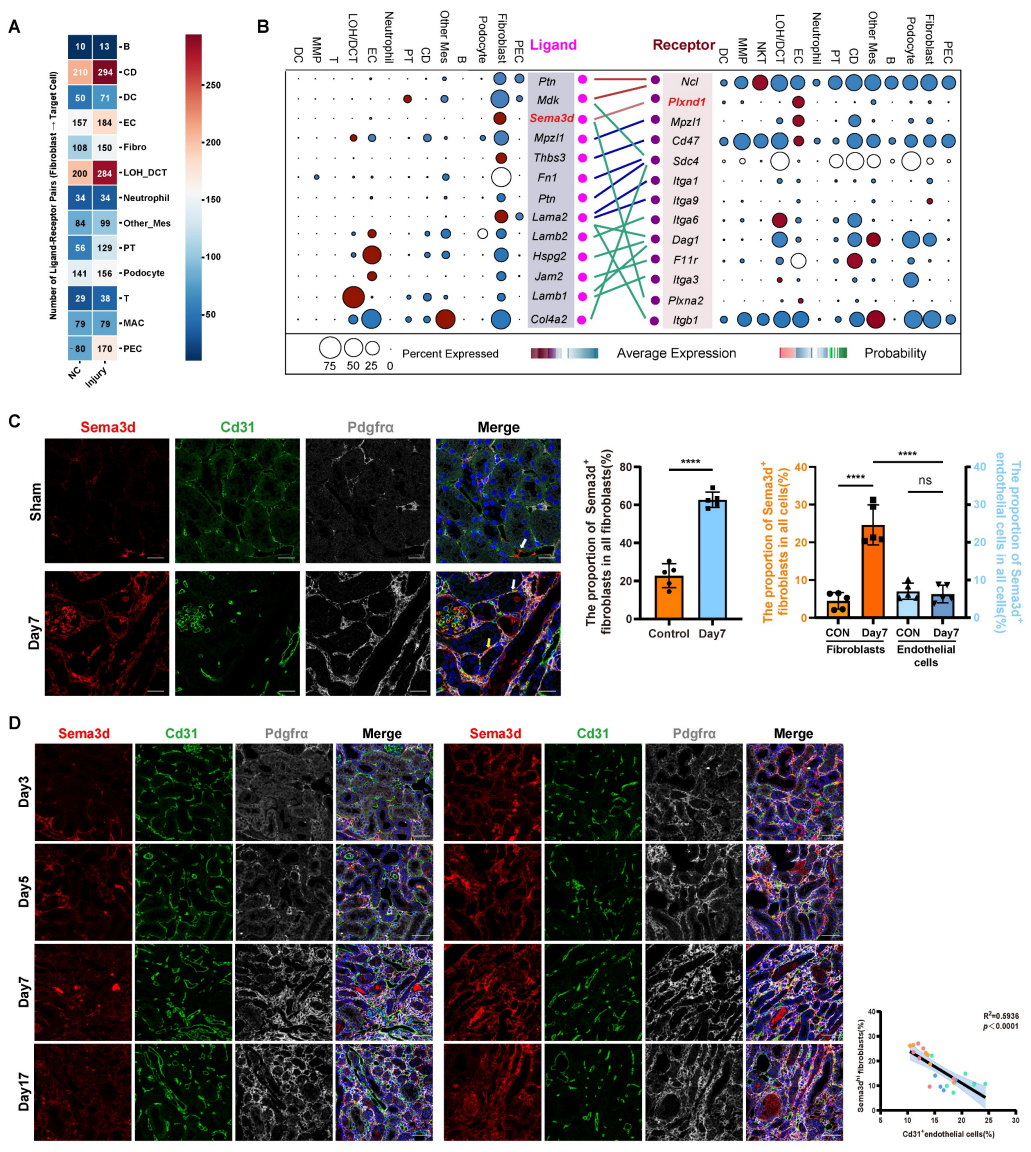
** Distribution of Sema3d^hi^ fibroblasts after uIRI in mice. (A)** Heatmap showing the effects of kidney fibroblasts on other types before and after uIRI. Values indicate interaction pairs. **(B)** Ligand-receptor pairs between renal fibroblasts and endothelial cells after uIRI. Left: ligands from fibroblasts; right: receptors on endothelial cells. Point size denotes the proportion of cells expressing the genes, while color indicates average expression levels across all cell types. **(C)** Immunofluorescence staining of Sema3d (red), Cd31 (green), and Pdgfrα (gray), in kidney tissues of control and uIRI mice. White arrows indicate Sema3d-expressing endothelial cells; yellow arrows indicate Sema3d^hi^ fibroblasts. Scale bar = 25 μm. Middle bar plot showing the proportion of Sema3d^hi^ fibroblasts among all fibroblasts; Right bar plot showing the proportion of Sema3d-expressing fibroblasts and endothelial cells among all interstitial cells. n = 5. ns: no significance, ****p < 0.0001. **(D)** Immunofluorescence staining of Sema3d (red), Cd31 (green), and Pdgfrα (gray)in kidneys on days 3, 5, 7, and 17 post-uIRI. Scale bar = 25 μm. Right scatter plot correlating Sema3d^hi^ fibroblast counts with Cd31^+^ endothelial cell counts on days 3 (blue dots), 5 (green dots), 7 (orange dots), and 17 (red dots) post-uIRI, indicating a negative correlation in distribution of Sema3d^hi^ fibroblasts and Cd31^+^ endothelial cells (R² = 0.5936, p < 0.0001).

To further investigate the impact of fibroblasts on other cells in the kidneys after uIRI, we performed cell-cell interaction analysis (Figure 2A). Compared to control group, the effects of fibroblasts on nearly all other cell compartments were enhanced after uIRI. Notably, the effects on LOH, CD tubular epithelial cells and endothelial cells were particularly pronounced, with a higher number of interaction pairs observed among these groups. While the interaction between tubular epithelial cells and fibroblasts has been extensively studied, the interaction pairs between fibroblasts and endothelial cells was notably increased and deserved further investigation. In the kidneys of uIRI mice, *Sema3d* and *Plxnd1* were specifically highly expressed by fibroblasts and endothelial cells, respectively, and the interaction between fibroblasts and endothelial cells was characterized by a robust Sema3d-Plexin D1 interaction (Figure 2B).

Through Cd31 staining of kidney tissue sections from sham and uIRI mice at 3, 5, 7, and 17 days post-surgery, we observed that Cd31-positive capillaries became increasingly rarefied with the chronic progression of kidney injury (Figure S3). Next, we aimed to investigate whether Sema3d^hi^ fibroblasts played a role in the capillary rarefaction observed in kidney tissues following IRI. To further explore the presence of Sema3d^hi^ fibroblasts, we performed triple immunofluorescence staining using anti-Sema3d, anti-Cd31, and anti-Pdgfrα antibodies on kidney tissues collected on the 7th day following either sham surgery or uIRI. In the tubulointerstitial areas of sham-operated kidneys, Sema3d^hi^ fibroblasts (Sema3d^+^/ Pdgrfα^+^) accounted for about 20% of the total fibroblast population (Pdgrfα^+^) and 4% of all tubulointerstitial cells. By the 7th day post-uIRI, the proportion of Sema3d^hi^ fibroblasts in the renal tubulointerstitial area significantly increased to about 60%, representing 25% of all cells, respectively. Additionally, some endothelial cells in the tubulointerstitial areas of both sham and uIRI kidneys were found to express Sema3d. However, the proportion of Sema3d^hi^ endothelial cells (Sema3d^+^/ Cd31^+^) among all tubulointerstitial cells did not exhibit significant change between sham and uIRI, remaining around 6% of the total tubulointerstitial cell population. Notably, the number of Sema3d^hi^ fibroblasts in the tubulointerstitial areas was approximately four times greater than that of Sema3d-expressing endothelial cells. These results indicate that the substantial increase in Sema3d expression after uIRI is primarily attributed to Sema3d^hi^ fibroblasts (Figure 2C). Triple immunofluorescence staining was also conducted on the 3rd, 5th, 7th, and 17th days after uIRI. We observed that Sema3d^hi^ fibroblasts (Sema3d^+^/Pdgfrα^+^) began to emerge in the injured kidney by day 3 post-uIRI and persisted throughout the chronic phase of AKI. In the areas with Sema3d^hi^ fibroblast expression, Cd31^+^ endothelial cells expression was greatly reduced. Statistically, a negative correlation was observed between the number of Sema3d^hi^ fibroblasts and the density of Cd31^+^ peritubular capillaries (PTC) in the same region of uIRI-exposed kidneys (Figure 2D).

### Sema3d^hi^-NRK49F inhibits endothelial cell migration and angiogenesis

**Figure 3:**
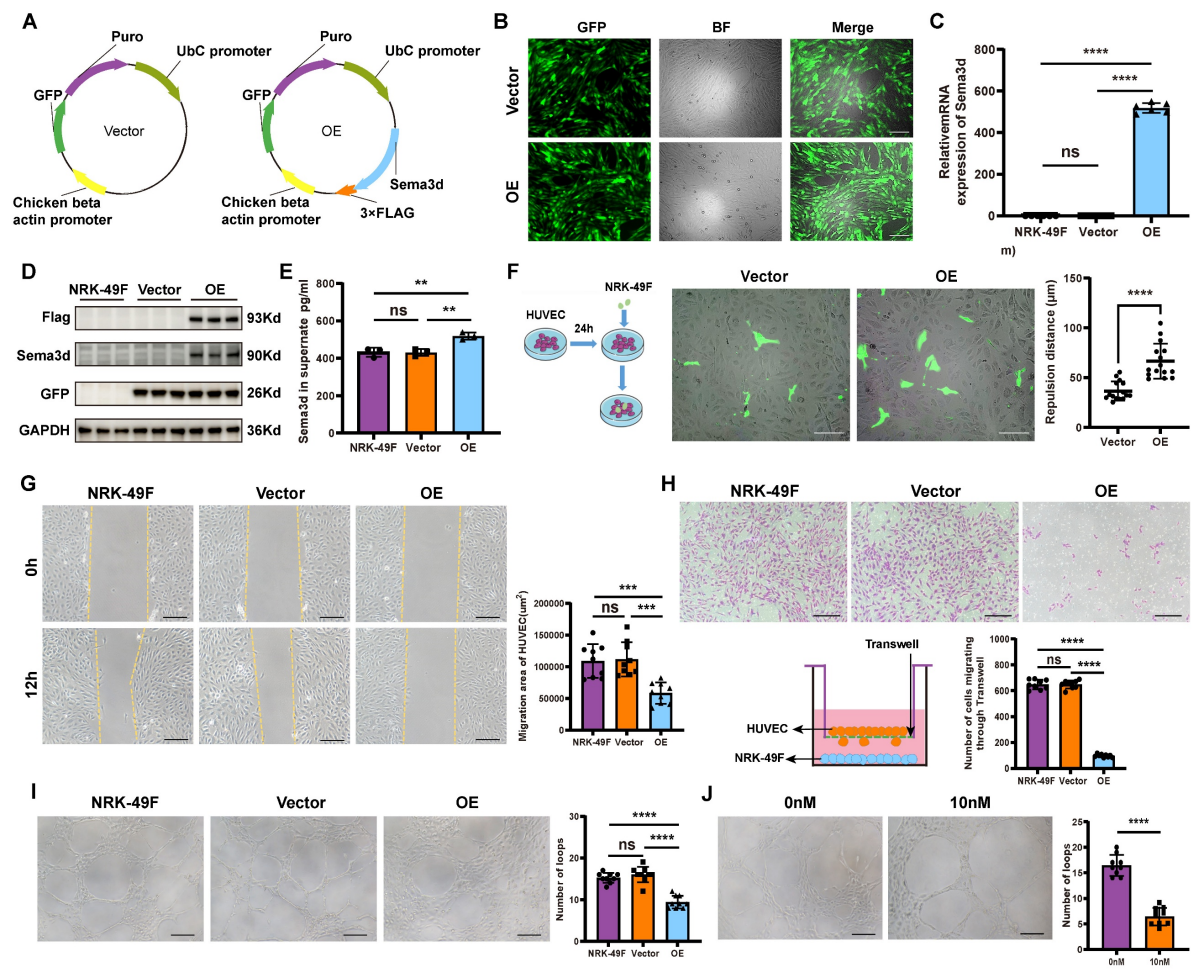
** Inhibition of HUVECs migration and angiogenesis by Sema3d^hi^-NRK49F cells. (A)** Schematic of the control vector and Sema3d overexpression plasmid. **(B)** Fluorescence images of NRK49F cells transfected with Lentiviruses carrying GFP (Vector) or Sema3d, Flag tag, and GFP (OE). Scale bar = 200 μm. **(C)** Relative *Sema3d* mRNA levels in NRK-49F, NRK49F-Vector and NRK49F-OE cells. n = 6. **(D)** Western blotting of Flag, Sema3d and GFP from NRK-49F, NRK49F-Vector and NRK49F-OE cells. n = 3. **(E)** Quantification of Sema3d level in the conditioned medium of NRK-49F, NRK49F-Vector and NRK49F-OE cells by ELISA. n = 3. **(F)** Repulsion assay between NRK49F-Vector, NRK49F-OE cells and HUVECs. Left: experimental flowchart; right: average distance statistics. Scale bar = 200 μm. n = 15. **(G)** Wound healing assay of HUVECs exposed to conditioned medium from NRK-49F, NRK49F-Vector and NRK49F-OE cells. Right: migration area statistics. Scale bar = 200 μm, n = 9. **(H)** Transwell migration assay of HUVECs co-cultured with NRK-49F, NRK49F-Vector and NRK49F-OE cells. Scale bar = 200 μm, n = 9. **(I)** Angiogenesis assay of HUVECs stimulated with conditioned medium from NRK-49F, NRK49F-Vector and NRK49F-OE cells. Scale bar = 200 μm, n = 9. **(J)** Angiogenesis assay of HUVECs stimulated with control medium and 10 nM Sema3d recombinant protein. Scale bar = 200 μm, n = 9. ns: no significance, ** p < 0.01, *** p < 0.001, **** p < 0.0001.

To investigate the functions of Sema3d^hi^ fibroblasts, NRK-49F cells overexpressing Sema3d (NRK49F-OE or Sema3d^hi^-NRK49F) and control vector (NRK49F-Vector) were generated (Figure S4 and Figure 3A). Fluorescence microscopy, rt-qPCR for *Sema3d* and western blot analyses of Flag, Sema3d and GFP confirmed the successful overexpression of exogeneous Sema3d in NRK-49F cells (Figure 3B-D). The ELISA assay detected a significant increase in the level of Sema3d in the Sema3d^hi^-NRK49F supernatant (Figure 3E). Bulk RNA sequencing was performed on NRK-49F, NRK49F-Vector and NRK49F-OE cell lines to assess whether altered *Sema3d* expression could influence fibroblast function. Validation through western blot, rt-qPCR and cell migration assay revealed that the proliferation capacity, collagen production, and pro-inflammatory function of Sema3d^hi^-NRK49F cells did not exhibit significant change, while the migration ability might be substantially enhanced (Figure S5).

The NRK49F-Vector and NRK49F-OE cells were seeded onto HUVEC monolayers, where significant repulsion of surrounding endothelial cells by OE group was observed (Figure 3F). In the wound healing assay, 12 hours after stimulating HUVECs with conditioned medium from NRK-49F, NRK49F-Vector, and NRK49F-OE cells, the scratch area of the OE group was significantly larger than that in the NRK-49F and vector groups, indicating that HUVEC migration was inhibited by the conditioned medium from Sema3d^hi^-NRK49F cells (Figure 3G). In the chemotactic migration assay, NRK-49F, NRK49F-Vector, and NRK49F-OE cells were seeded in the lower chamber of a Transwell, and HUVECs were placed in the upper chamber for co-culture over 72-96 hours. We observed that co-culturing with the OE group significantly reduced the number of HUVECs that migrated through the Transwell compared to the NRK-49F and vector groups, indicating that HUVEC chemotactic migration was inhibited (Figure 3H). Furthermore, HUVECs resuspended in the conditioned medium from NRK-49F, NRK49F-Vector, and NRK49F-OE cells were seeded onto matrix gel. After 6 hours, we observed that the number of vascular structures formed by HUVECs in the OE group was significantly lower than that in the NRK-49F and vector groups (Figure 3I). Similarly, the number of vascular structures formed by HUVECs in the 10nM Sema3d recombinant protein treated group was also significantly reduced compared to the control group (Figure 3J). These findings indicate that the conditioned medium from Sema3d^hi^-NRK49F cells inhibits endothelial cell angiogenesis, mirroring the effect of Sema3d recombinant protein. Collectively, the above results suggest that Sema3d^hi^-NRK49F cells inhibit endothelial cell migration and angiogenesis through the secretion of Sema3d.

### Sema3d^hi^-NRK49F cells reduces the formation of focal adhesions and promotes cytoskeletal collapse in endothelial cells

**Figure 4:**
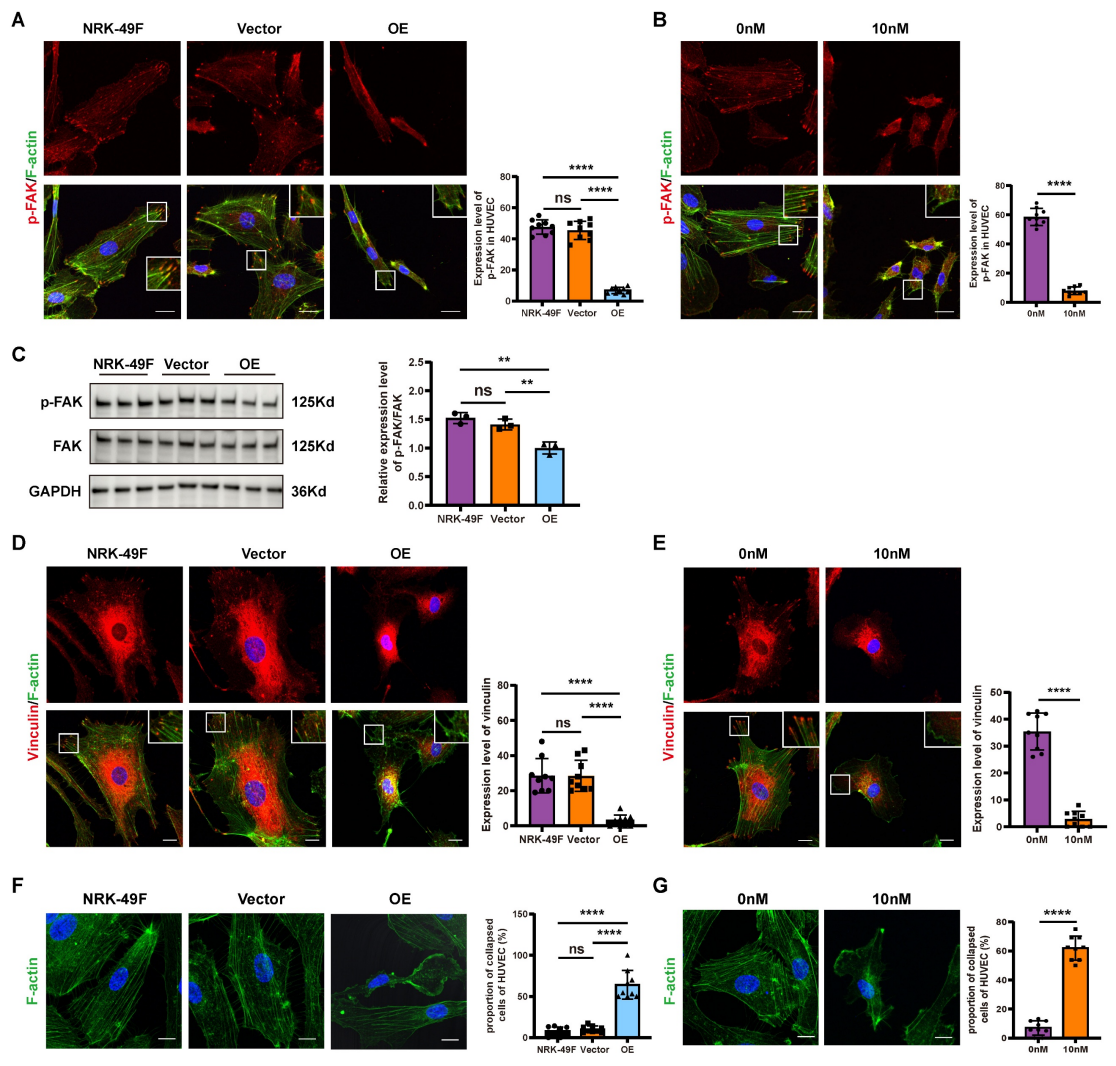
** Effects of Sema3d^hi^-NRK49F cells on endothelial cell focal adhesions and cytoskeleton. (A)** Immunofluorescence staining of p-FAK in HUVEC stimulated with conditioned medium from NRK-49F, NRK49F-Vector and NRK49F-OE cells. Scale bar = 20 μm. n = 9. **(B)** Immunofluorescence staining results of p-FAK in HUVEC stimulated by control medium and 10nM Sema3d recombinant protein. Scale bar = 20 μm. n = 9. **(C)** Western blot detection of p-FAK in HUVEC stimulated by the conditioned medium from NRK-49F, NRK49F-Vector and NRK49F-OE cells and quantification results. n = 3. **(D)** Immunofluorescence staining of vinculin in HUVEC stimulated by the conditioned medium from NRK-49F, NRK49F-Vector and NRK49F-OE cells. Scale bar = 20 μm. n = 9. **(E)** Immunofluorescence staining of vinculin in HUVEC stimulated by control medium and 10nM Sema3d recombinant protein. Scale bar = 20 μm. n = 9. **(F)** Immunofluorescence staining of F-actin (cytoskeleton) in HUVEC stimulated by the conditioned medium from NRK-49F, NRK49F-Vector and NRK49F-OE cells. Scale bar = 20 μm. n = 9. **(G)** Immunofluorescence staining of F-actin (cytoskeleton) in HUVEC stimulated by control medium and 10nM Sema3d recombinant protein. Scale bar = 20 μm. n = 9. ns: no significance, **p < 0.01, ****p < 0.0001.

Pseudopodia and focal adhesions play crucial roles in driving and guiding cell migration. The phosphorylation of FAK and the subsequent activation of vinculin significantly influence the formation and stabilization of pseudopodia and focal adhesions during this process 34-36. Following stimulation of HUVECs with the conditioned medium from NRK-49F, NRK49F-Vector, and NRK49F-OE cells, we observed that the phosphorylation level of FAK in HUVECs stimulated by the OE group conditioned medium was markedly decreased compared to the NRK-49F and vector groups (Figure 4A and C). Additionally, vinculin levels were reduced, and a significant collapse of cytoskeleton was noted (Figure 4D and F). Similarly, stimulation of HUVECs with Sema3d recombinant protein resulted in reduced phosphorylation levels of FAK, decreased vinculin levels, and cytoskeletal disintegration when compared to the control group (Figure 4 B, E and G). These findings suggest that Sema3d^hi^-NRK49F cells modulate the focal adhesion dynamics and cytoskeletal architecture of endothelial cells through the secretion of Sema3d.

### Sema3d^hi^-NRK49F cells induce integrin internalization and Arf6 activation in endothelial cells

**Figure 5:**
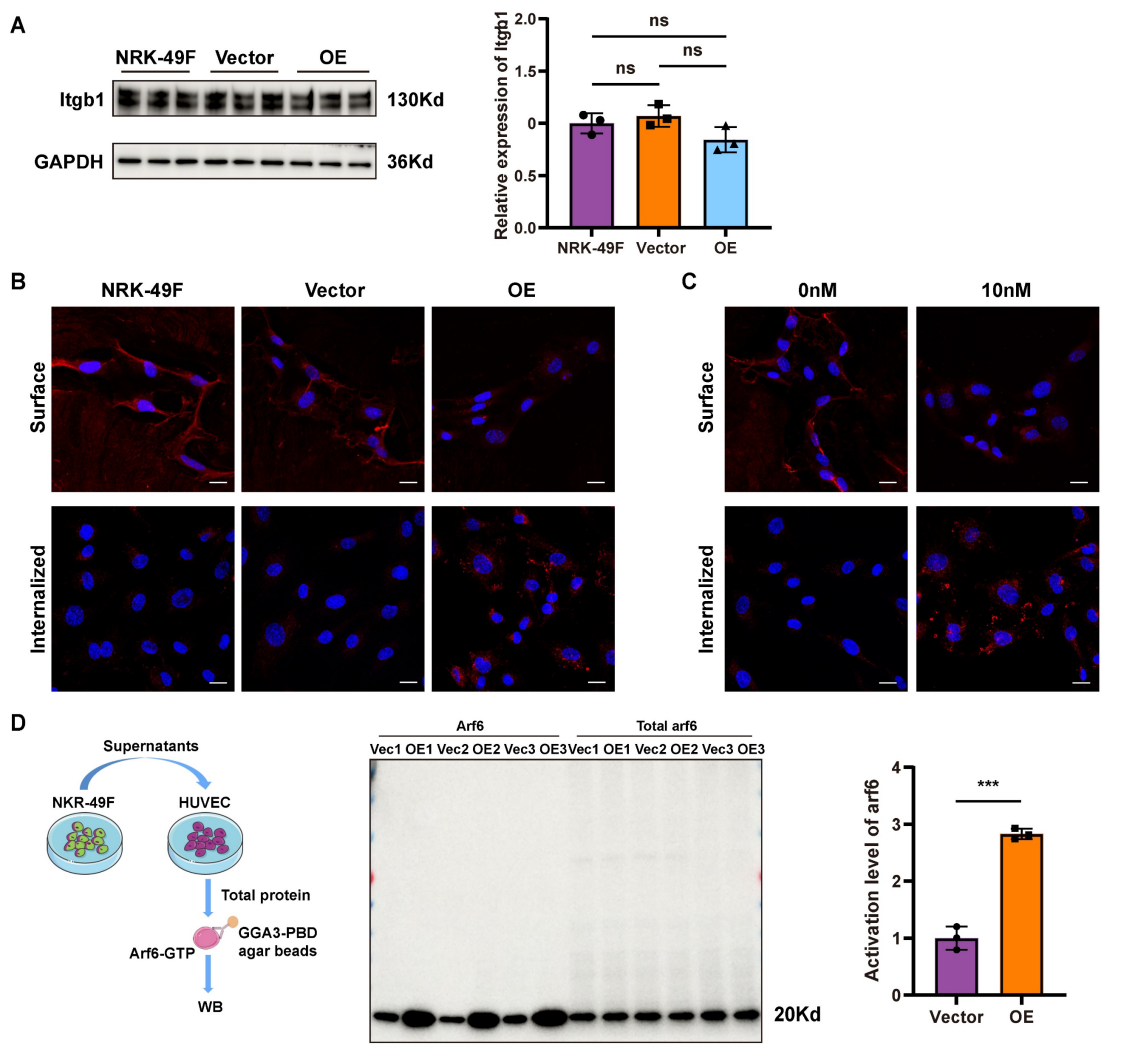
** Effects of Sema3d^hi^-NRK49F on integrin internalization and Arf6 activation in endothelial cells. (A)** Western blot detection of integrin β1 in HUVEC stimulated with conditioned medium from NRK-49F, NRK49F-Vector and NRK49F-OE cells and quantification results. n = 3. **(B)** Immunofluorescence staining of integrin β1 in HUVEC stimulated by the conditioned medium from NRK-49F, NRK49F-Vector and NRK49F-OE cells. Scale bar = 20 μm. **(C)** Immunofluorescence staining of integrin β1 in HUVEC stimulated by control medium and 10nM Sema3d recombinant protein. Scale bar = 20 μm.** (D)** Effects of conditioned medium fromNRK49F-Vector and NRK49F-OE cells on the activation of Arf6 in HUVEC. The left shows the pull-down experiment flowchart, the middle shows the western blot results, and the right provides statistical results, n = 3. ns: no significance, ***p < 0.001.

Integrin β1 is a membrane receptor that plays a pivotal role in cell migration by facilitating the activation of downstream FAK 34, 36. To detect whether Sema3d^hi^-NRK49F cells affect the expression of integrin β1 in HUVECs, HUVECs were stimulated with conditioned medium from NRK-49F, NRK49F-Vector, and NRK49F-OE cells, followed by western blot for Integrin β1. The results showed that the expression level of integrin β1 in HUVECs did not change significantly (Figure 5A). However, in integrin internalization assay, stimulation of HUVECs with the conditioned medium from Sema3d^hi^-NRK49F cells resulted in a significant decrease in the distribution of integrin β1 on the cell membrane, accompanied by an increase in its intracellular localization (Figure 5B). Similarly, after stimulation with 10nM Sema3d recombinant protein, a decrease in the cell membrane distribution of integrin β1 was observed in HUVECs, along with an increase in its intracellular distribution compared to the control group (Figure 5C). These findings indicate that Sema3d^hi^-NRK49F cells can promote the internalization of integrins in endothelial cells by secreting Sema3d.

Arf family proteins play a crucial role in the remodeling of actin cytoskeleton, thus mediating the transport of substances between the plasma membrane and endosomes. Notably, Arf6 has been implicated in the regulation of integrins internalization and cytoskeleton morphology 37, 38. Pull-down experiments were conducted using GGA3-PBD agarose beads to detect the activation level of Arf6 in HUVECs. Control experiments to detect Arf6 total protein and active form of Arf6-GTP and inactive form of Arf6-GDP was performed first (Figure S6). To determine whether Sema3d^hi^-NRK49F can activate Arf6 in HUVECs, HUVECs were stimulated with conditioned medium from NRK49F-Vector and NRK49F-OE groups, respectively. The results demonstrated a significant increase in the activation level of Arf6 in HUVECs exposed to the OE group conditioned medium compared to the vector group (Figure 5D), indicating that Sema3d^hi^-NRK49F can activate Arf6 and induce membrane integrin internalization in HUVECs.

### Sema3d^hi^-NRK49F cells inhibit endothelial cell migration by secreting Sema3d and acting on Plexin D1 receptors in endothelial cells

**Figure 6:**
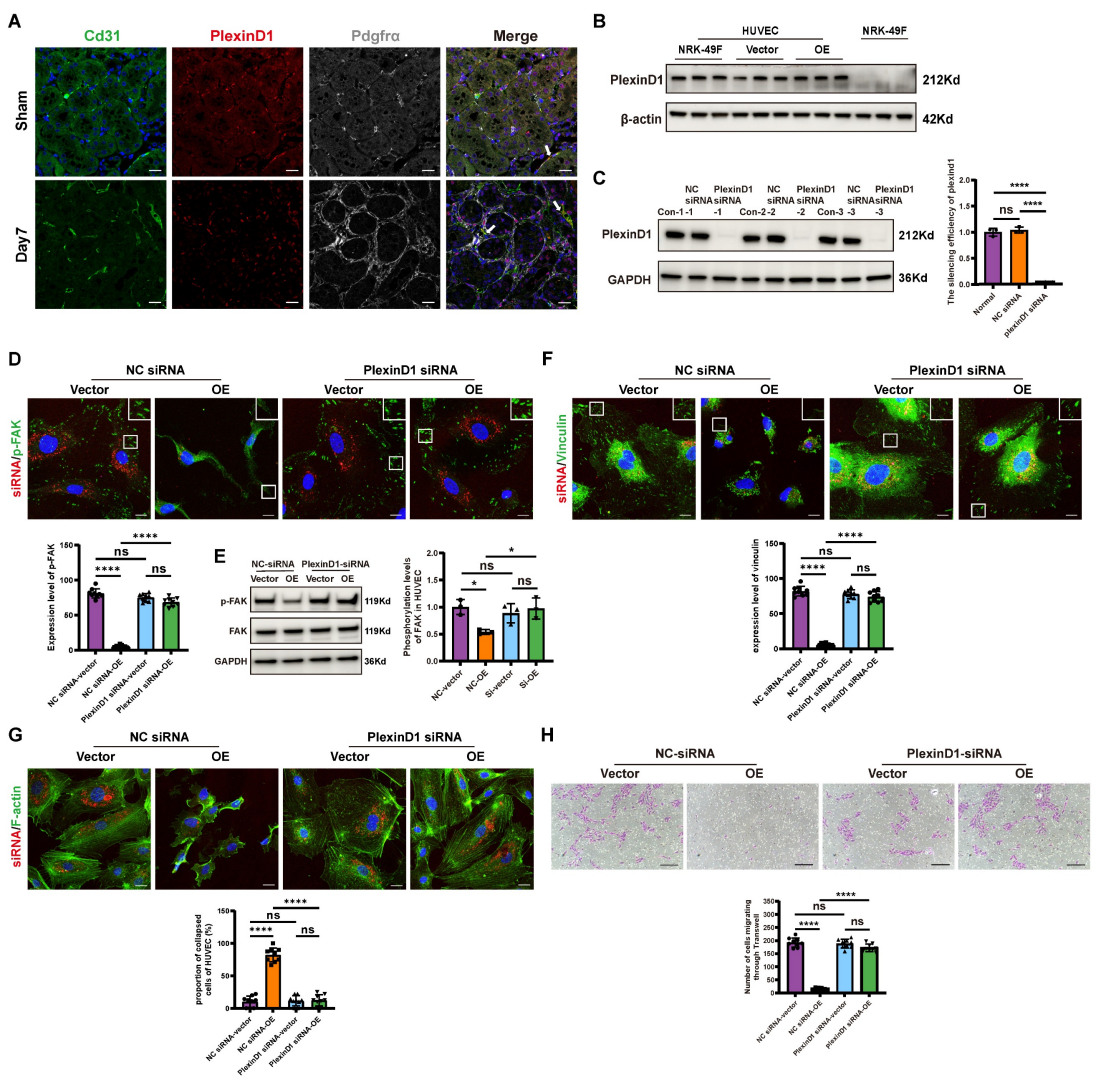
** Sema3d secreted by Sema3d^hi^-NRK49F cells affects endothelial cell migration via Plexin D1 receptor. (A)** Immunofluorescence staining of Cd31 (green), Plexin D1 (red), and Pdgfrα (white) in mouse kidneys pre- and post-uIRI. The white arrows indicate Cd31^+^/Plexin D1^+^/Pdgfrα^-^ cells. Scale bar = 20 μm. **(B)** Western blot detection of Plexin D1 in HUVEC and NRK-49F cells. n = 3.** (C)** Western blot detection of Plexin D1 demonstrating successful knockdown of Plexin D1 in HUVECs by siRNA, with statistical results shown. n = 3. **(D)** Immunofluorescence staining of p-FAK in HUVEC stimulated by the conditioned medium from NRK49F-Vector and NRK49F-OE cells after control siRNA and Plexin D1 siRNA treatment. Scale bar = 20 μm, n = 9. **(E)** Western blot detection of p-FAK expression in HUVEC stimulated by the conditioned medium from NRK49F-Vector and NRK49F-OE cells after control siRNA and Plexin D1 siRNA treatment. n = 3. **(F)** Immunofluorescence staining of vinculin in HUVEC stimulated by the conditioned medium from NRK49F-Vector and NRK49F-OE cells after control siRNA and Plexin D1 siRNA treatment. Scale bar = 20 μm, n = 9. (**G**) Immunofluorescence staining of F-actin in HUVEC stimulated by the conditioned medium from NRK49F-Vector and NRK49F-OE cells after control siRNA and Plexin D1 siRNA treatment. Scale bar = 20 μm, n = 9. **(H)** Chemotactic migration experiment of HUVEC stimulated by the conditioned medium from NRK49F-Vector and NRK49F-OE cells after control siRNA and Plexin D1 siRNA treatment. Scale bar = 200 μm, n = 9. ns: no significance, *p < 0.05, ****p < 0.0001.

Previous cell-cell interaction analysis indicated that Sema3d-Plexin D1 was an important ligand-receptor pair mediating the effects of fibroblasts on endothelial cells (Figure 2B). It has been reported that the binding of ligands to Plexin D1 can activate downstream Arf6 signaling pathways 39. Through triple immunofluorescence staining, we confirmed that Plexin D1 was predominantly expressed in the endothelial cells (Plexin D1^+^/Cd31^+^) of the uIRI mouse kidney (Figure 6A). Similarly, Plexin D1 was found to be expressed in HUVECs, but was nearly absent in NRK-49F cells (Figure 6B).

To further investigate the role of Plexin D1, we knocked down its expression in HUVECs (Figure 6C), and then stimulated these cells with conditioned medium from NRK49F-Vector and NRK49F-OE group cells. Compared to the vector group, stimulation of control HUVECs with the OE group conditioned medium resulted in a significant decrease in phosphorylation level of FAK, a reduction in vinculin, and a collapse of the cytoskeleton. However, these effects were not observed in HUVECs with Plexin D1 knocked down (Figure 6D-G). Furthermore, following co-culture with NRK49F-OE cells, the number of HUVECs that migrated through the Transwell was significantly reduced compared to the vector group, indicating an inhibition of chemotactic migration. In contrast, no significant change in the number of HUVECs passing through the Transwell was observed when Plexin D1-knockdown HUVECs were co-cultured with NRK49F-OE cells (Figure 6H). These results indicate that Sema3d^hi^-NRK49F cells inhibit endothelial cells migration by secreting Sema3d, which acts on the Plexin D1 receptor in endothelial cells.

### Knockdown of Sema3d by AAV9-Sema3d shRNA ameliorates kidney fibrosis *in vivo*

**Figure 7:**
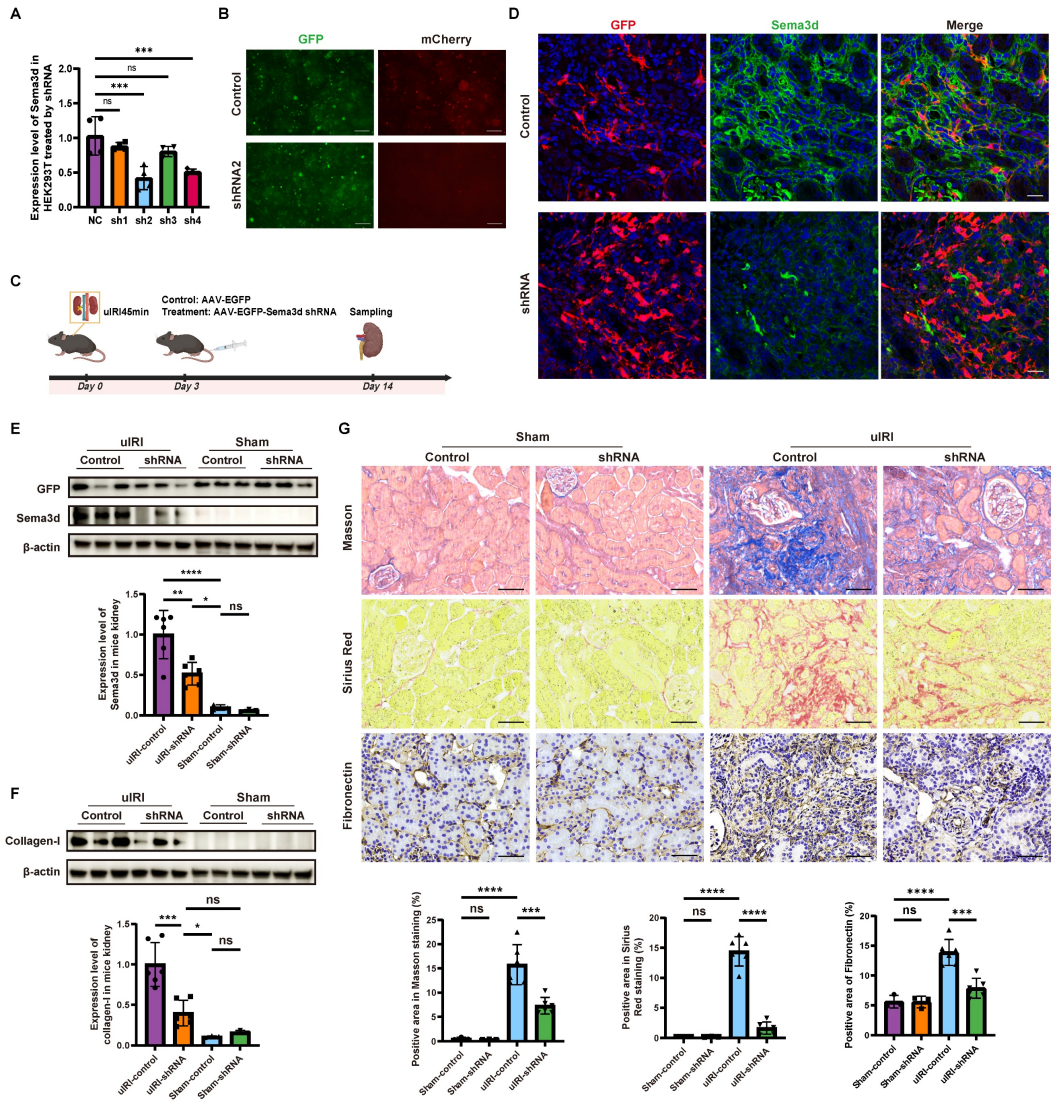
** Knockdown of Sema3d by AAV9-Sema3d shRNA mitigates renal fibrosis in uIRI mice. (A)** Knockdown efficiency of different Sema3d-shRNA sequences on mSema3d overexpression in HEK293 cells. **(B)** Sema3d knockdown by control and Sema3d-shRNA2 in HEK293 cells. Green fluorescence indicates the expression of control and Sema3d-shRNA2, and red fluorescence represents the overexpression level of mSema3d.** (C)** Flow chart on protocol of AAVs injection in mice post-uIRI surgery and kidney sample collection. **(D)** Immunofluorescence staining of GFP (red) and Sema3d (green) in kidney tissues of uIRI mice injected with control or Sema3d-shRNA. Scale bar = 25 μm. **(E)** Western blot detection of Sema3d knockdown efficiency in mice. **(F)** Collagen I expression in the kidneys of uIRI or sham-operated mice with AAV injection. **(G)** Representative images of Masson's trichrome staining, Sirius red staining, and Fibronectin immunohistochemistry in the kidneys of uIRI or sham-operated mice with AAV injection. Sham operated group n=3, uIRI operated group n=6. ns: no significance, *p < 0.05, **p < 0.01, ***p < 0.001, ****p < 0.0001.

We engineered five plasmids, including ssAAV.CAG.EGFP.miR30shRNA 1-4 (mSema3d).WPRE3.SV40 for knockdown and pcDNA3.1(+)-PGK.mCherry-P2A-mSema3d(NM_028882.4).bGHpA for overexpression. Each Sema3d-shRNA plasmid was co-transfected with the overexpression plasmid into HEK293 cells. The knockdown efficiency was assessed via rt-qPCR and immunofluorescence detection, revealing that shRNA2 exhibited the most significant knockdown of Sema3d expression (Figure 7A and B).

To further explore the role of Sema3d *in vivo*, we constructed an AAV9-based delivery system for Sema3d-shRNA. On day 3 post-uIRI surgery, AAV9-EGFP-Sema3d shRNA and AAV9-EGFP control viruses were injected into the tail veins of mice at a dose of 5×10^11^ GCs per mouse. Kidney tissues were harvested 14 days post-surgery, during the peak phase of fibrosis (Figure 7C).

Immunofluorescence staining indicated that Sema3d expression was significantly elevated in the control virus-treated group, where it co-localized with GFP antibody staining. Conversely, in the AAV9-EGFP-Sema3d shRNA intervention group, Sema3d expression was markedly reduced in areas expressing GFP (Figure 7D). Western blot analysis corroborated these findings, showing weak Sema3d protein expression in the sham groups, with a significant increase noted in the uIRI group treated with control virus. In contrast, the uIRI group administered the Sema3d shRNA virus displayed a pronounced reduction in Sema3d protein levels (Figure 7E).

Further analysis of the fibrosis marker Collagen-1 revealed weak expression in both the sham and shRNA intervention groups, while significantly elevated levels were observed in the control virus-treated uIRI group. The intervention with AAV9-EGFP-Sema3d shRNA significantly reduced Collagen-1 expression in uIRI kidney tissues (Figure 7F). Additionally, histological analysis using Masson's trichrome, Sirius Red, and Fibronectin immunohistochemical staining confirmed that fibrosis markers were significantly elevated in the control virus-treated group following uIRI, whereas they were significantly diminished in the shRNA intervention group (Figure 7G).

### Existence of SEMA3D^+^ fibroblasts in human kidney

**Figure 8:**
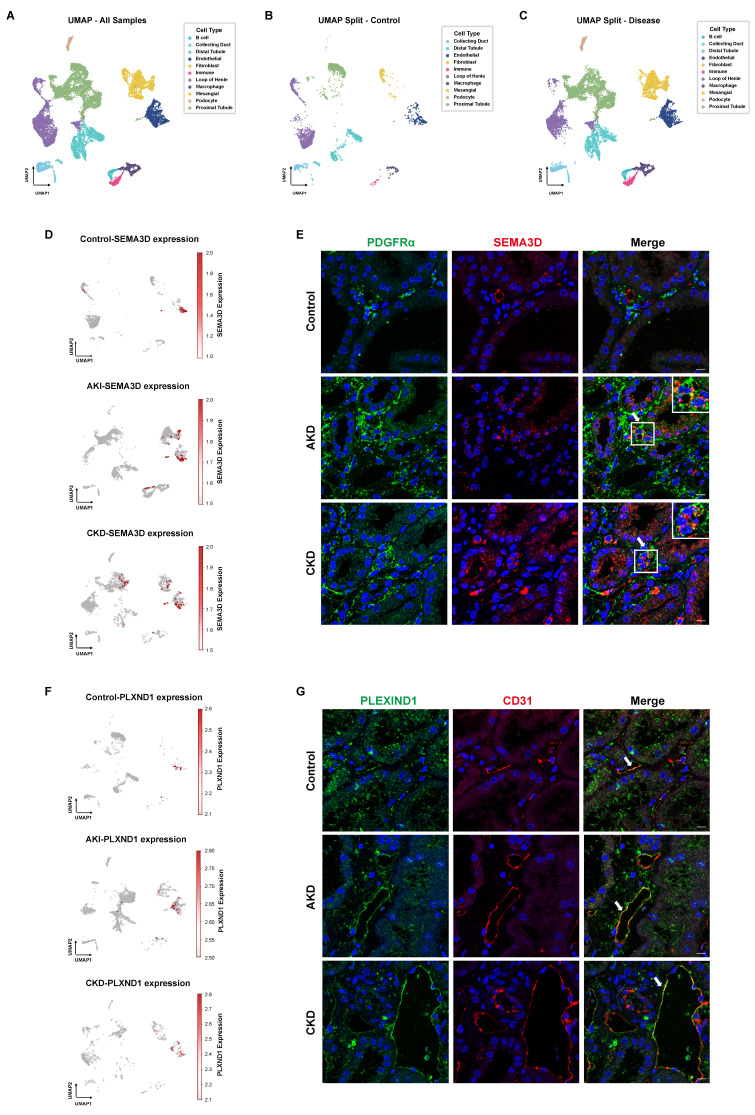
** Presence of SEMA3D^+^ fibroblasts and expression of PLEXIND1 in human kidneys. (A)** UMAP plot of scRNA-seq data from six human kidney samples obtained from KPMP website. **(B)** UMAP plot of scRNA-seq data from 2 healthy control human kidney samples from KPMP.** (C)** UMAP plot of scRNA-seq data from 4 AKI and CKD patient kidney samples from KPMP. **(D)** Feature plots of *SEMA3D* expression on UMAP plots from control, AKI and CKD human samples. **(E)** Immunofluorescence staining of PDGFRα (green) and SEMA3D (red) on kidney samples from healthy control and patients with AKD or CKD.** (F)** Feature plots of *PLXND1* expression on UMAP plots from control, AKI and CKD human samples.** (G)** Immunofluorescence staining of PLEXIND1 (green) and CD31 (red) on kidney samples from healthy control and patients with AKD or CKD.

To further validate the presence of SEMA3D^+^ fibroblasts, we analyzed human scRNA-seq data obtained from the KPMP website. Our analysis comprised four disease samples (AKI and hypertensive CKD) and two healthy control samples. After performing batch correction, dimensionality reduction, clustering, and cell type annotation based on characteristic gene expression, we generated a UMAP that encompassed all kidney cell types (Figure 8A-C). In the control group, *SEMA3D* was predominantly expressed in endothelial cells. Conversely, in the AKI and CKD patient groups, *SEMA3D* expression was detected in fibroblasts and, to some extent, in tubular epithelial cells in CKD (Figure 8D). Notably, the *PLXND1* gene was primarily expressed in endothelial cells (Figure 8F).

Additionally, we conducted immunofluorescence staining on kidney tissue sections from patients diagnosed with AKD and CKD, as well as from a normal donor kidney. The staining results revealed that in the tubulointerstitial regions, SEMA3D colocalized with the fibroblast marker PDGFRα, confirming the presence of SEMA3D^+^ fibroblasts in the kidney tissues of patients (Figure 8E). Moreover, PLEXIND1 colocalized predominantly with the endothelial cell marker CD31, indicating the presence of PLEXIND1^+^ endothelial cells within the patient kidney tissues (Figure 8G).

## Discussion

Microvascular damage leading to capillary rarefaction is a critical step in the progression of AKI to CKD. Typically, microvasculature responds to injury by generating new vessels, thereby facilitating self-repair. The repair process following microvascular injury involves the degradation of the basement membrane, detachment of pericytes, and loosening of endothelial cell junctions, resulting in increased microvascular permeability, plasma protein extravasation, and the formation of a temporary matrix layer. Endothelial cells adhere to the extracellular matrix via integrins, allowing for migration, proliferation, and extension to form lumens. This process subsequently attracts pericytes to deposit onto the basement membrane, promoting the maturation and stabilization of microvessels 9. However, compared to renal tubules, the regenerative capacity of renal microvasculature is relatively limited 18, 19. This limitation is attributed to the low proliferative ability of endothelial cells and their susceptibility to EndoMT 20-22. Additionally, the rapid proliferation of fibroblasts, which are widely present in the stroma following injury, warrants further investigation regarding their impact on angiogenesis.

Several studies have demonstrated the heterogeneity of fibroblasts following AKI 40-43. However, previous research primarily characterized fibroblast populations using immunohistochemical methods that rely on the recognition of specific markers (such as FAP^hi^ and α-SMA^hi^ fibroblasts) 43. This approach has limited comprehensive analysis of the gene expression profiles and functions of those subpopulations, making it difficult to identify new subpopulations under disease states. The rapid advancement of scRNA-seq technology in recent years has provided a more powerful tool for exploring cellular heterogeneity. Consequently, we established a comprehensive scRNA-seq database from whole kidneys of uIRI mouse model. By analyzing the gene expression profiles of the whole kidney cells before and after IRI, we defined mesenchymal partition as those expressing high levels of *Pdgfrb* and/or *Pdgfra*, as well as *Col1a1* and/or *Acta2*, and further identified 6 distinct fibroblast subpopulations. Among those, the C1-Tcf21^hi^, C2-Fut9^hi^, and C4-Sfrp4^hi^ subpopulations were resident renal fibroblasts enriched in GO terms related to tubular epithelial cell promotion and angiogenesis, suggesting a potential reparative role following kidney injury. The C3-Ltbp2^hi^ subpopulation, which arisen after injury, resembled typical myofibroblasts and was rich in contractile and pro-fibrotic genes. This subpopulation was abundantly present in the late stages of injury, continuously secreting large amounts of ECM, leading to excessive matrix deposition and causing deformation of the interstitial connective tissue through contraction. In contrast, the C0-Sema3d^hi^ subpopulation, which constituted the largest proportion of fibroblasts after kidney injury, was primarily enriched in functions related to migration, inflammation regulation, and inhibition of angiogenesis. Trajectory analysis and immunofluorescence staining results suggest that this fibroblast subpopulation may represent a transitional state from fibroblasts to myofibroblasts.

By analyzing cell-cell interaction between fibroblasts and other cells in the kidneys of uIRI mice, we found that the interaction of fibroblasts with endothelial cells is significantly enhanced after injury. The ligand-receptor pair between *Sema3d* and *Plxnd1* was particularly notable, with *Sema3d* predominantly expressed by fibroblasts and *Plxnd1* primarily expressed by endothelial cells. Immunofluorescence staining for Sema3d, Pdgfrα, and CD31 in kidney tissues from mice at day 7 post-uIRI revealed that, compared to the sham-operated group, the proportion of endothelial cells expressing Sema3d did not show significant changes after uIRI injury. In contrast, the number of Sema3d^hi^ fibroblasts significantly increased among tubulointerstitial cells, indicating that Sema3d becomes predominantly enriched in fibroblasts following kidney injury. As a member of the Semaphorin (SEMA) family, Sema3d is a secreted protein primarily studied for its roles in the development of the nervous and cardiovascular systems, as well as in tumor biology, where it serves as a repulsive signaling molecule guiding neuronal axons and vascular pattern 11, 44. Although previous studies have reported the expression of Sema3d in cancer-associated fibroblasts (CAFs) 45, there is a lack of relevant research in the context of renal pathology.

Endothelial cell migration plays a crucial role in angiogenesis. During the formation of new blood vessels, endothelial cells must migrate to the surface of the ECM and move and proliferate in an orderly manner, guided by various environmental factors, to accurately construct the new vascular network 9. Cell migration is a dynamic and complex multi-step process that involves the extension of the cell front, the transformation of adhesion complexes, the generation of traction forces, and the retraction and separation of the cell rear. Adhesion complexes consist of various proteins, such as integrins (e.g., α5β1, α2β1), kinases (e.g., FAK, Src), scaffold proteins (e.g., paxillin, talin, vinculin), and actin stress fibers, which play a driving role in cell migration. When integrins bind to the ECM, they undergo conformational changes and clustering, which initiate intracellular signaling pathways, including the autophosphorylation of FAK, thereby promoting the maturation of adhesion complexes and the reorganization of the cytoskeleton 34-36. Consequently, the expression of integrins on the cell membrane is a critical factor in initiating cell migration. Integrins can be internalized through clathrin-mediated or caveolin-mediated endocytosis and transported to endosomes for processing, after which they may be recycled back to the cell membrane or degraded. The small GTPase Arf6 plays a crucial role in the internalization of integrins and the regulation of the cytoskeleton 46, 47. The activity of Semaphorin (SEMA) family proteins is mediated by Plexin receptors, with Neuropilins (NRPs) and receptor tyrosine kinases also serving as co-receptors that work together with Plexins to activate signaling pathways 13. Research has shown that Plexin D1 can activate Arf6 in pancreatic ductal adenocarcinoma cells 48. Our bioinformatics analysis indicates that the interaction between *Sema3d* and *Plxnd1* plays a significant role in the communication between fibroblasts and endothelial cells. Therefore, we hypothesize that Sema3d secreted by fibroblasts may inhibit endothelial cell migration through its interaction with endothelial Plexin D1.

We established a Sema3d-overexpressing NRK-49F cell line and observed a significant increase in Sema3d levels in its culture supernatant. Following stimulation with the conditioned medium from Sem3d^hi^-NRK49F cells and exogenous recombinant Sema3d, we noted a significant decrease in phosphorylation level of FAK, a substantial reduction in vinculin, and a marked collapse of the cytoskeleton. Additionally, the intracellular distribution of Integrin β1 increased while its presence on the cell membrane decreased, impairing HUVECs' ability to effectively adhere and migrate. Further investigations revealed that Sema3d secreted by Sema3d^hi^-NRK49F cells significantly activates Arf6 in HUVECs, which may contribute to the internalization of integrins in these cells. Using immunofluorescence staining, we examined the distribution of the Plexin D1 receptor in mouse kidneys and found that Plexin D1 is predominantly located on endothelial cells, with no detectable expression in fibroblasts. After knocking out Plexin D1 in HUVECs, we observed that under stimulation with the conditioned medium from Sema3d^hi^-NRK49F cells, there were no significant changes in the p-FAK and vinculin expression levels or the cytoskeleton. The ability of HUVECs to migrate through Transwells was also not significantly inhibited by the Sema3d^hi^-NRK49F cell conditioned medium. These findings indicate that Sema3d secreted by Sema3d^hi^ fibroblasts indeed suppresses endothelial cell migration through its interaction with the Plexin D1 receptor. However, in the narrow space of the tubulointerstitial region *in vivo*, both fibroblasts and endothelial cells exhibit strong morphological plasticity, so it is also possible that these two cell types may make direct contact, inhibiting endothelial cell migration through the Sema3d-Plexin D1 receptor interaction.

AAV9-EGFP-Sema3d shRNA intervention significantly reduced the expression of fibrosis markers in the kidneys of mice following uIRI. Furthermore, the presence of SEMA3D^+^ fibroblasts was confirmed by analyzing human scRNA-seq data and through immunofluorescence staining of kidney sections from patients with AKD and CKD. In these samples, increased level of SEMA3D was observed in fibroblasts, contrasting with healthy controls, where it was primarily localized to endothelial cells. These *in vivo* results suggest that Sema3d play a pivotal role in the progression of kidney fibrosis, and targeted knockdown of this gene may represent a promising therapeutic strategy for alleviating renal fibrosis. Additionally, these findings provide strong evidence for the existence of SEMA3D^+^ fibroblasts in human kidney tissue under disease conditions. However, further research is necessary to elucidate their relationship with kidney diseases and their potential roles in disease progression.

## Conclusion

**Figure 9:**
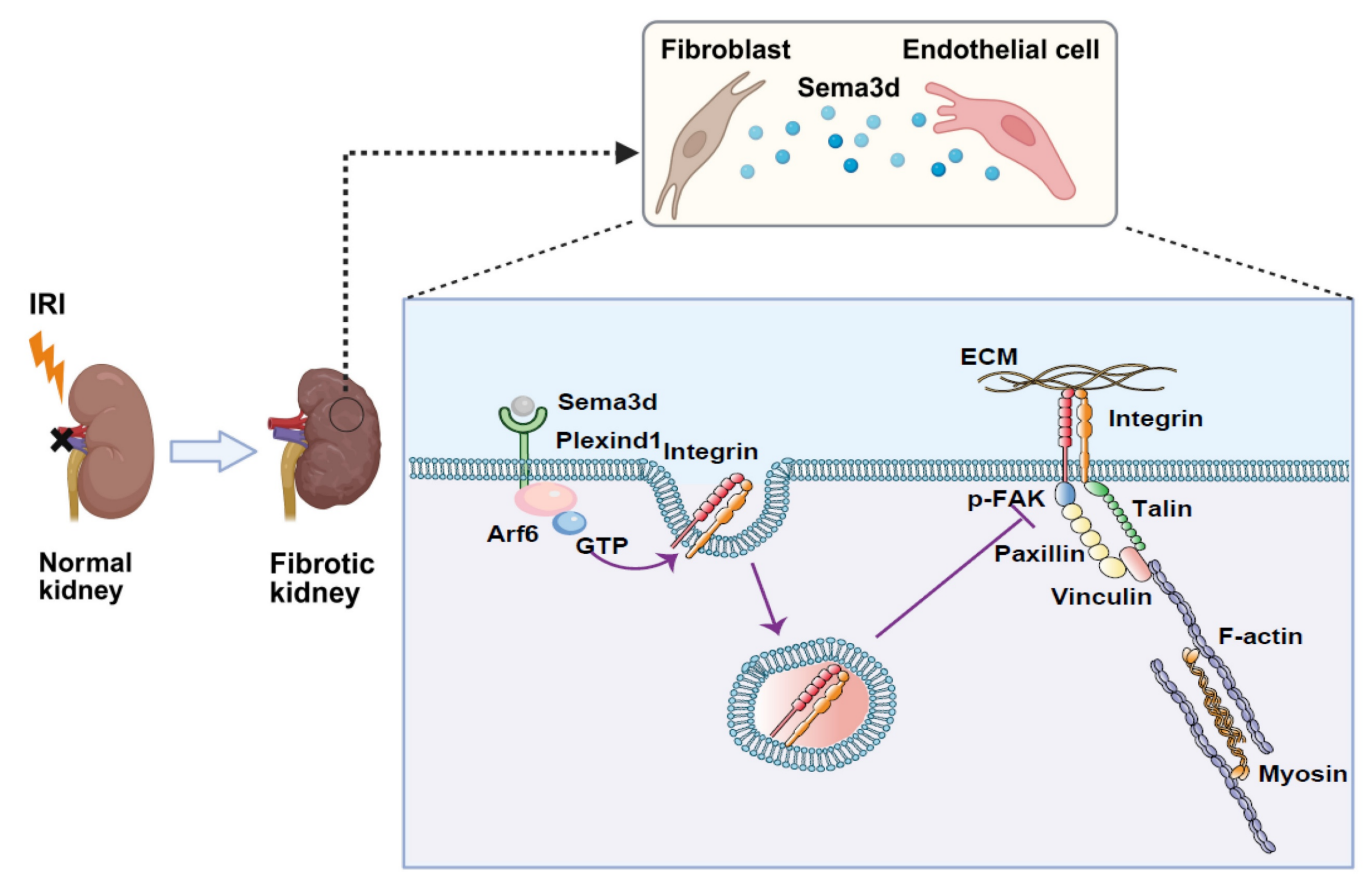
** Mechanisms by which Sema3d^hi^ fibroblasts regulate endothelial cell migration and angiogenesis.** A subpopulation of fibroblasts secretes high levels of Sema3d, which binds to Plexin D1 receptors on endothelial cells. This binding activates the small GTPase Arf6 in endothelial cells, resulting in increased internalization of integrins. The internalized integrins inhibit filopodia formation and cause cytoskeletal collapse, blocking endothelial cell migration and angiogenesis. This process contributes to the loss of renal microvasculature, accelerating the progression from AKI to CKD. (The Figure was partially created using BioRender.)

This study investigates the heterogeneity of fibroblasts and the functional characteristics of fibroblast subpopulations during the transition from AKI to CKD by analyzing scRNA-seq data from uIRI mice. We identified a subpopulation of fibroblasts that express high levels of *Sema3d*, which can secrete Sema3d and interact with the endothelial cell-specific Plexin D1 receptors. This interaction activates the small GTPase Arf6 in endothelial cells, leading to increased internalization of integrins. Consequently, this process inhibits filopodia formation and induces cytoskeletal collapse, thereby obstructing endothelial cell migration and angiogenesis. This, in turn, exacerbates the loss of renal microvasculature and promotes the progression from AKI to CKD (Figure 9). Our findings provide new potential targets for mitigating renal fibrosis and microvascular loss following AKI, suggesting that intervention in the Sema3d signaling pathway may serve as an effective strategy to prevent the transition from AKI to CKD.

## Supplementary Material

Supplementary figures, table headings, and table S3.

Supplementary table S1.

Supplementary table S2.

Supplementary table S4.

Supplementary table S5.

Supplementary table S6.

## Abbreviations

AKI: acute kidney injury
CKD: chronic kidney disease
uIRI: unilateral ischemia reperfusion injury
HUVEC: human umbilical vein endothelial cell
ECM: extracellular matrix
EndoMT: endothelial to mesenchymal transition
scRNA-seq: single-cell RNA sequencing
AAV9: adeno-associated virus serotype 9
GC: genome copy
