# Supplementary material for: Sema3dhi Fibroblasts Promote Acute Kidney Injury Fibrotic Progression Through Confining Endothelial Cell Migration
Source: Int J Biol Sci. 2026 Jan 1;22(3):1283–305. doi: 10.7150/ijbs.124971 (PMC12837656; doi:10.7150/ijbs.124971)
Supplement: Supplementary file 1 — Supplementary figures, table headings, and table S3. [file ijbsv22p1283s1.pdf]

Supplemental Figures

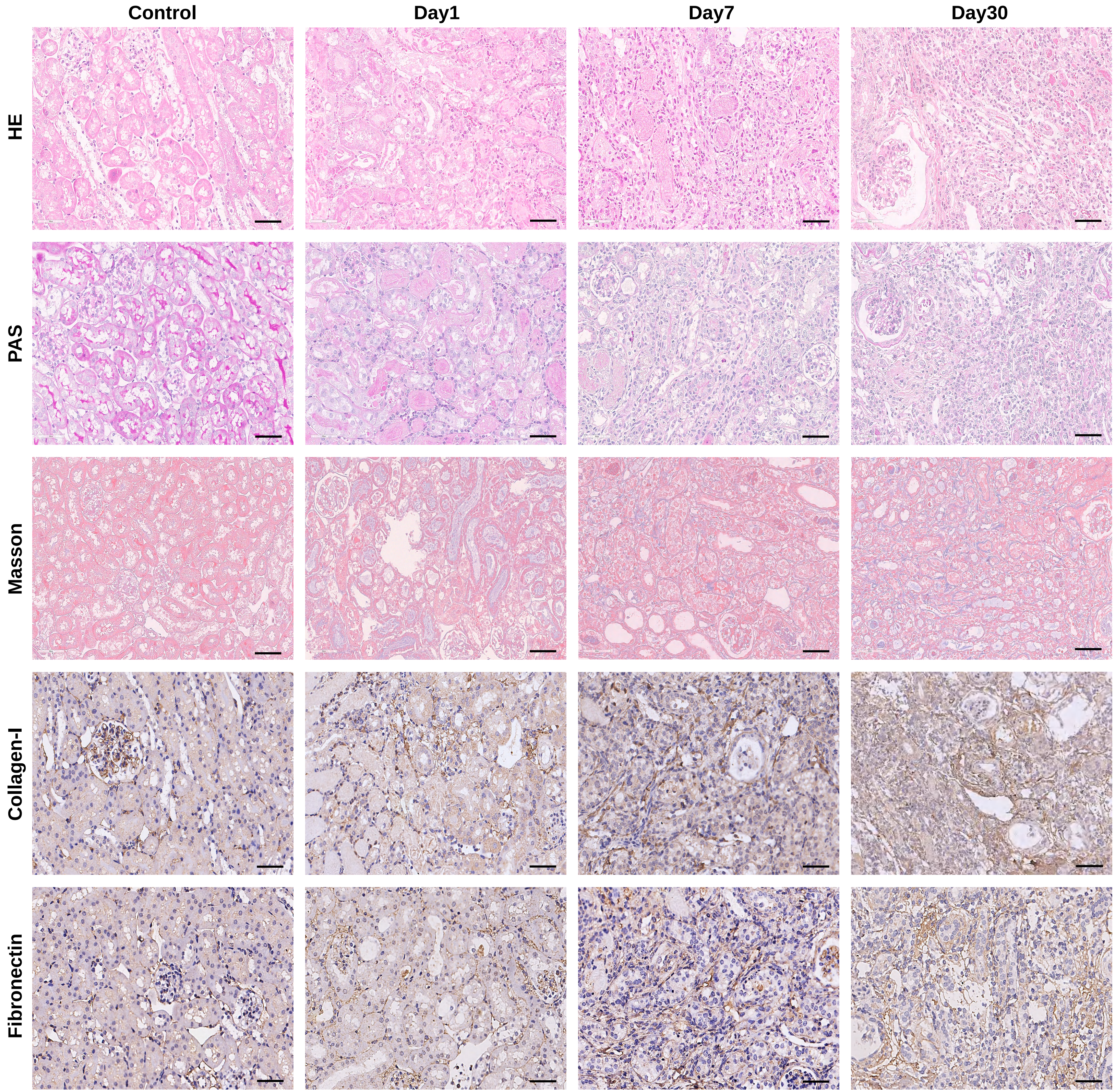

Figure S1. Representative histopathological images of renal tissue from uIRI mice underwent hematoxylin and eosin (HE), periodic acid-Schiff (PAS), Masson's trichrome, Collagen I and Fibronectin staining. Scale bar = 60μm.

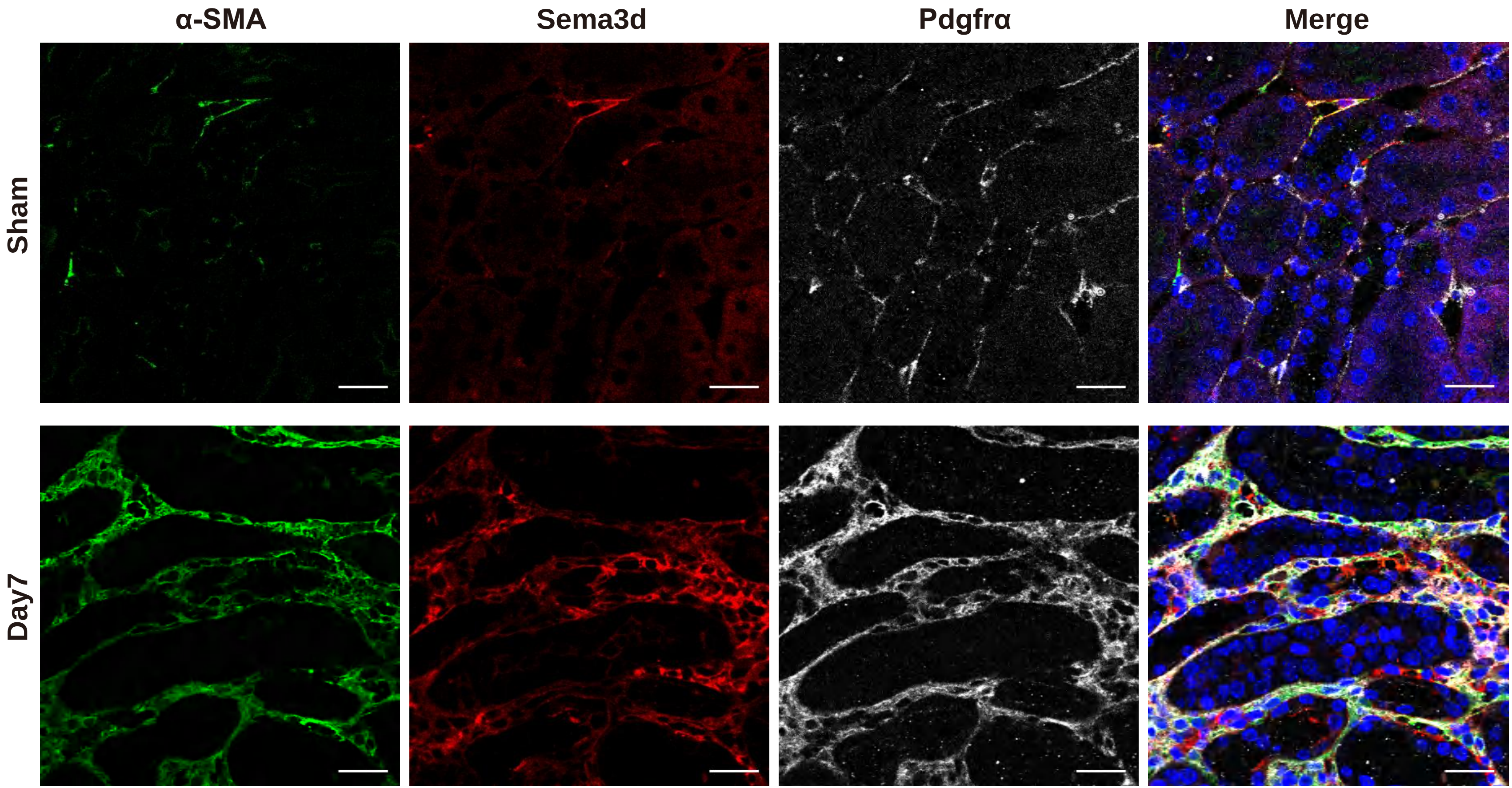

Figure S2. Colocalization of Sema3d⁺ fibroblast with myofibroblast marker α-SMA. Scale bar = 25μm.

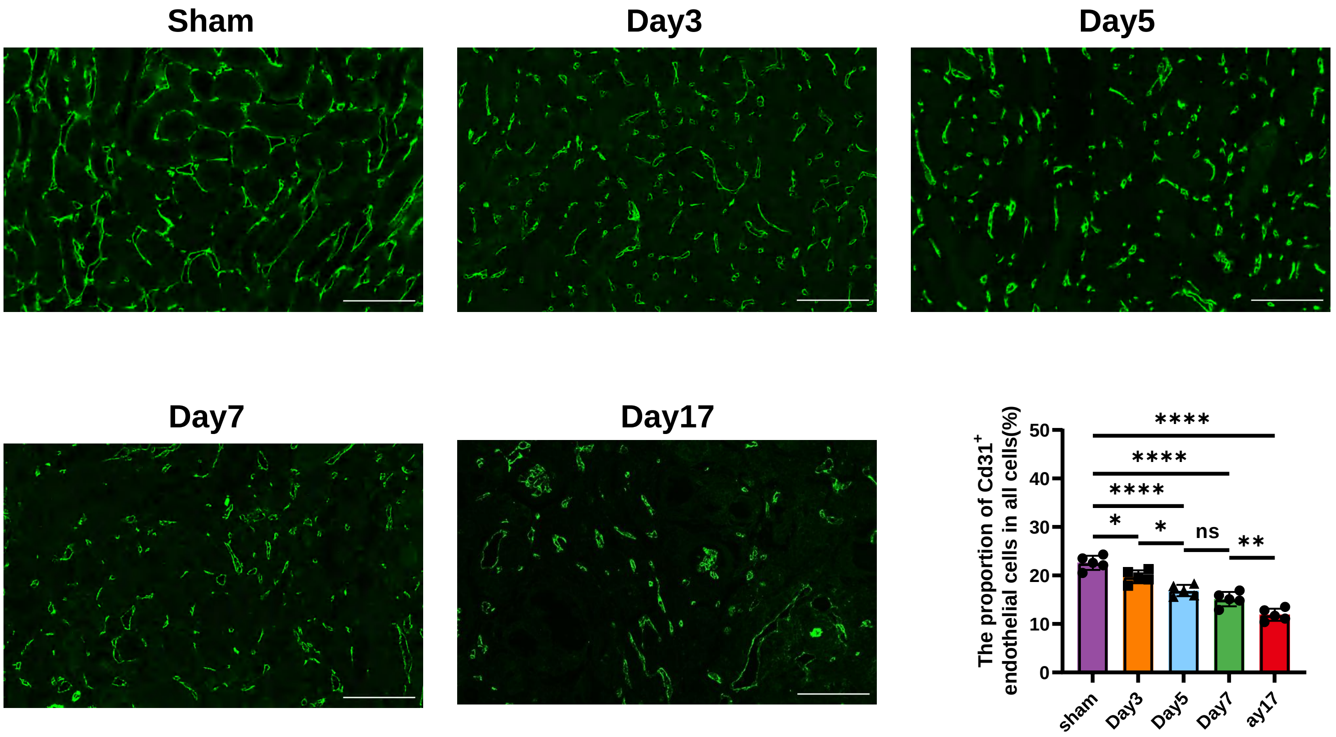

Figure S3. Dynamic changes of peritubular capillaries (Cd31^{+}) after uIRI. Scale bar = 100μm.

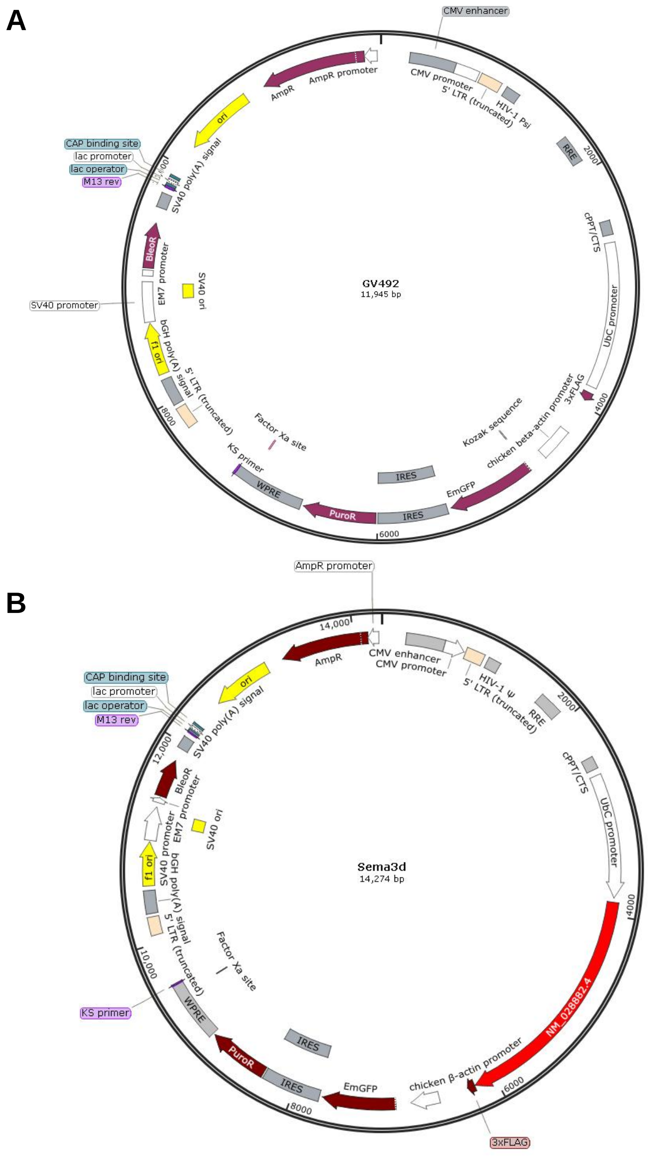

Figure S4. Schematic diagram of the plasmid structure. (A) Schematic diagram of the structure of vector plasmid. (B) Schematic diagram of the structure of Sema3d-overexpressing plasmid.

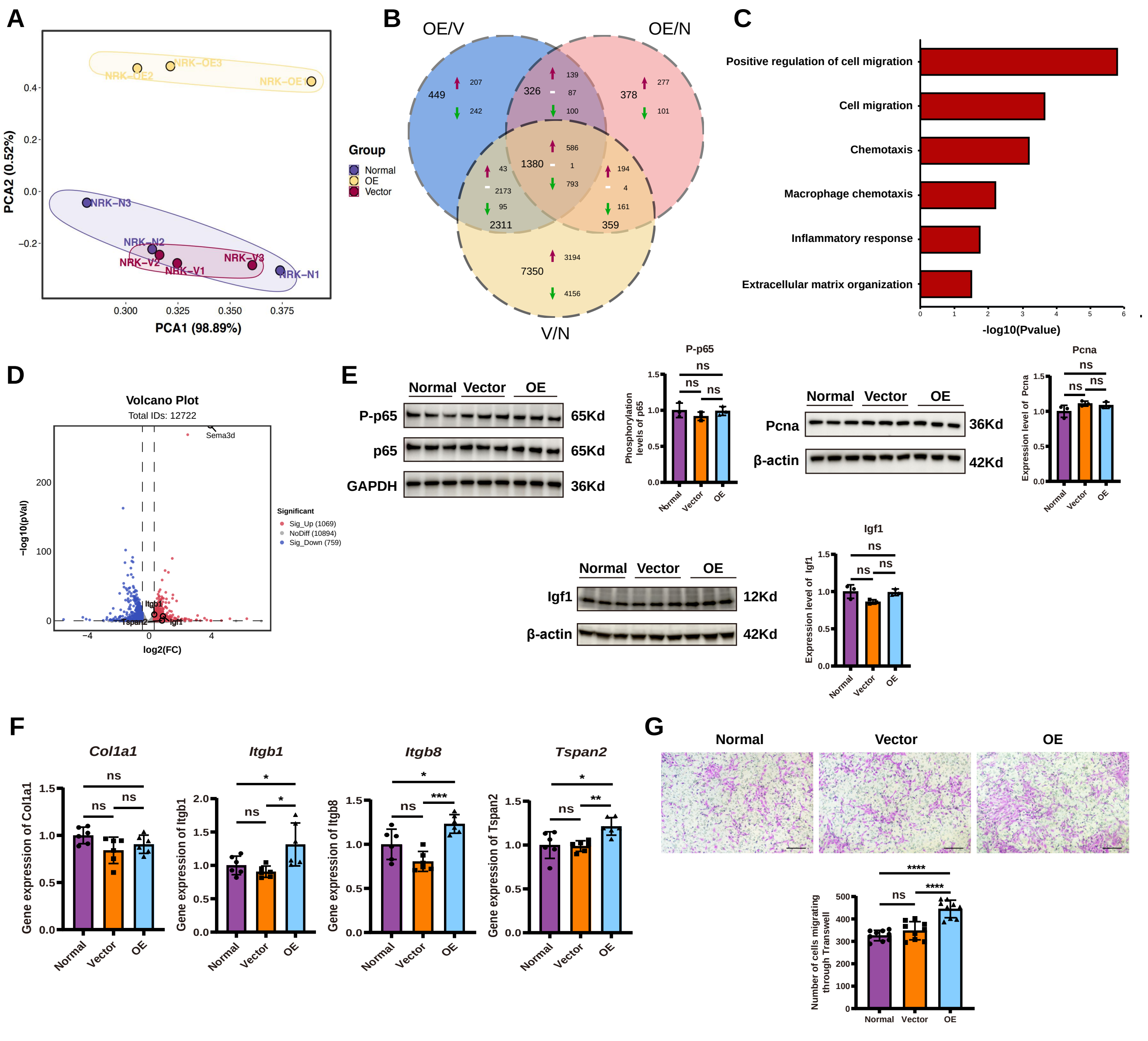

Figure S5. The influence of Sema3d overexpression on fibroblast function. (A) PCA plot of bulk RNA-seq data from Normal NRK-49F, NRK49F-Vector, and NRK49F-OE cells. (B) Venn diagram of DEGs among Normal NRK-49F, NRK49F-Vector, and NRK49F-OE cells. (C) GOBP enrichment analysis of bulk RNA-seq data from NRK49F-OE cells. (D) Volcano plot of DEGs between NRK49F-OE and NRK49F-Vector groups. (E) Western blot analysis of representative DEGs and phenotypic marker proteins among NRK-49F, NRK49F-OE and NRK49F-Vector groups, with quantitative analysis below. n = 3. (F) qPCR validation of mRNA levels for some DEGs and phenotypic marker genes. n = 6. (G) Transwell migration assay of NRK49F-OE cells. Scale bar = 200µm. n = 9. ns: no significance, * $p < 0.05$, ** $p < 0.01$, *** $p < 0.001$, **** $p < 0.0001$.

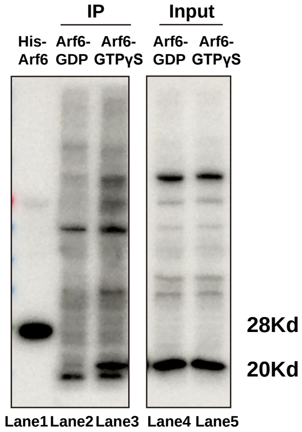

Figure S6. Pilot experiment for Arf6 activation assay. Lane 1: His-Arf6 control protein (5 ng) included in the assay kit. Lane 2: HUVEC cell lysates (500 µg) treated with GDP and pulled down using GGA3-PBD beads (negative control). Lane 3: HUVEC cell lysates (500 µg) treated with GTPγS and pulled down using GGA3-PBD beads (positive control). Lane 4: Total Arf6 from HUVEC cell lysates (20 µg) loaded with GDP. Lane 5: Total Arf6 from HUVEC cell lysates (20 µg) loaded with GTPγS.

Supplemental Tables:

Table S1. rt-qPCR primers.

Supplemental File (Excel)

Table S2. Top 10 DEGs in KPMP patient atlas.

Supplemental File (Excel)

Table S3. Patient information for SEMA3D and PLEXIND1staining.

Patient information for SEMA3D and PLEXIND1staining

Patient No.	Age	Biopsy BUN (mmol/L)	Biopsy Scr (μmol/L)	Biopsy eGFR (mL/min/1.73m²)	AKD or CKD
**1**	59	8.5	164	29.3	AKD
**2**	63	15.6	269.7	20.5	CKD

Table S4. Top 100 DEGs in 26 clusters of whole kidney cells.

Supplemental File (Excel)

Table S5. Top 10 DEGs in 6 clusters of mesenchymal cells.

Supplemental File (Excel)

Table S6. Top 100 DEGs in 6 clusters of fibroblasts.

Supplemental File (Excel)
